# Sex-specific typologies of older adults’ sedentary behaviors and their associations with health-related and socio-demographic factors: a latent profile analysis

**DOI:** 10.1186/s12877-021-02011-5

**Published:** 2021-01-19

**Authors:** Sofie Compernolle, Ilse De Bourdeaudhuij, Greet Cardon, Delfien Van Dyck

**Affiliations:** Department of Movement and Sports Sciences, Faculty of Medicine and Health Sciences, Ghent University, Watersportlaan 2, B-9000 Ghent, Belgium

**Keywords:** Sitting time, Sedentary time, Cluster analysis, Patterns, Mental health, Physical health

## Abstract

**Background:**

Some types of sedentary behaviors tend to cluster in individuals or groups of older adults. Insight into how these different types of sedentary behavior cluster is needed, as recent research suggests that not all types of sedentary behavior may have the same negative effects on physical and mental health. Therefore, the aim of this study was to identify sex-specific typologies of older adults’ sedentary behavior, and to examine their associations with health-related and socio-demographic factors.

**Methods:**

Cross-sectional data were collected as part of the BEPAS Seniors, and the Busschaert study among 696 Flemish older adults (60+). Typologies of self-reported sedentary behavior were identified using latent profile analysis, and associations with health-related and sociodemographic factors were examined using analyses of variances.

**Results:**

Five distinct typologies were identified from seven sedentary behaviors (television time, computer time, transport-related sitting time, sitting for reading, sitting for hobbies, sitting for socializing and sitting for meals) in men, and three typologies were identified from six sedentary behaviors (television time, transport-related sitting time, sitting for reading, sitting for hobbies, sitting for socializing and sitting for meals) in women. Typologies that are characterized by high television time seem to be related to more negative health outcomes, like a higher BMI, less grip strength, and a lower physical and mental health-related quality-of-life. Typologies that are represented by high computer time and motorized transport seem to be related to more positive health outcomes, such as a lower body mass index, more grip strength and a higher physical and mental health-related quality-of-life.

**Conclusions:**

Although causal direction between identified typologies and health outcomes remains uncertain, our results suggests that future interventions should better focus on specific types of sedentary behavior (e.g. television time), or patterns of sedentary behavior, rather than on total sedentary behavior.

**Supplementary Information:**

The online version contains supplementary material available at 10.1186/s12877-021-02011-5.

## Background

Older adults’ sedentary behavior (e.g. television viewing, reading, motorized transport) has been associated with decreased functional fitness and falls [1, 2], increased risk for many chronic diseases (metabolic syndrome [3, 4], type 2 diabetes [5], cardiovascular disease [6] and cancer [7]) and premature mortality [4, 8]. This is a reason for concern, as older adults are the most sedentary age group [9]. An accelerometer study – conducted in the UK – has shown that almost 70% of older adults are sedentary for more than 8.5 h of their waking day [10, 11]. Importantly, this sedentary time is spent in a variety of contexts for different purposes [12, 13]. Whereas television time, having meals, and reading have been identified as being the most prevalent types of sedentary behavior in both sexes (respectively, 47.9, 22.9 and 10.8% of total sedentary behavior) [14], the prevalence of other types of sedentary behavior seems to vary widely by sex [15]. For example it turns out that older men are five times more likely to have driven a car during the past week than older women, and that older men spend almost twice as much time on the computer than older women [15].

Just like with other health behaviors, different types of sedentary behavior do not occur in isolation from one another, and have shared correlates (e.g. age has been shown to be a shared positive correlate of household sedentary behavior and leisure time sedentary behavior; and alcohol consumption has been shown to be a shared positive correlate of leisure time sedentary behavior and transport-related sedentary behavior [16]). These shared correlates also seem to vary by sex [17–19]. Consequently, it can be expected that certain types of sedentary behaviors tend to cluster in the same men or women. To the best of our knowledge, sex-specific clustering of older adults’ sedentary behaviors has never been investigated. Nevertheless, this information is paramount to help developing interventions, as research suggests that not all types of sedentary behavior may have the same negative effects on physical and mental health [14, 20, 21]. Whereas strong evidence is available for the detrimental physical and mental health effects of passive sedentary behaviors (e.g. television viewing) [22, 23], the adverse health outcomes of cognitively demanding, and social sedentary behaviors are less clear. In fact, recent research has suggested that the latter sedentary behaviors could even produce beneficial health effects – especially in older adults [20, 22, 24]. These beneficial health effects are mainly expected on mental and cognitive health. For example, the cognitive demand of certain sedentary behaviors, such as reading and chess/cards-playing, might reduce cognitive decline, and the social aspect of other sedentary behaviors, such as going to a restaurant and having a coffee, might benefit mental health and quality of life [24]. In contrast to the potential beneficial effects on mental, and cognitive health, none of the sedentary behaviors is expected to positively influence physical health. All sedentary behaviors are characterized by low muscular unloading within the large skeletal muscle groups of the legs, back and trunk region, which initiates a cascade of harmful cellular events, such as hyperglycemia and hyperlipidemia [25]. Nevertheless, prior studies suggested that the negative effects on physical health also vary between different sedentary behaviors. This can probably be explained by the fact that some types of sedentary behavior are strongly related to other health risk behaviors, such as smoking and unhealthy dietary habits [26, 27].

As the number of older adults is expected to increase dramatically in the next decades, interventions that counter the negative health effects associated with sedentary behaviors, without damaging the positive health effects associated with related sedentary behaviors, are highly needed to safeguard older adults’ quality-of-life. However, most previous studies have investigated the associations of only one specific sedentary behavior with mental and physical health outcomes among older adults [21, 24, 28, 29].

Consequently, the first aim of this study is to identify sex-specific typologies of older adults’ sedentary behavior. The second aim is to examine the association between sex-specific typologies and health outcomes. Both physical (Body Mass Index [BMI], waist circumference, muscle strength, and physical health-related quality of life [QOL]) and mental (mental health-related QOL) health outcomes will be included. The third and final aim is to investigate the association between sex-specific typologies and socio-demographic characteristics. This is useful to decide on the specific population subgroups that should be targeted in future sedentary behavior interventions.

## Methods

### Study design

For this study, we combined cross-sectional data from two observational studies conducted within our research group with a similar methodology. The first study was the Belgian Environmental Physical Activity Study in Seniors (BEPAS Seniors) that was conducted between 2010 and 2012. The BEPAS seniors study was led by IDB and DVD, and aimed to gain insight into the physical environmental correlates of older adults’ activity-related behaviors [30]. The second study was conducted by Busschaert and colleagues and led by IDB, in 2013, to examine socio-ecological correlates of older adults’ domain-specific sedentary behavior [31]. Both studies were approved by the Ethics Committee of the Ghent University Hospital (B670201423000 and B670201317406, respectively) and all participants provided written informed consent.

### Recruitment and participants

#### BEPAS seniors

Stratified cluster sampling was used to select 20 neighborhoods in Ghent (i.e. city in Flanders, Belgium) and suburbs. The neighborhood sampling process has been described in detail elsewhere [30]. Briefly, the neighborhoods were selected based on their walkability (high vs. low), and their neighborhood annual household income (high vs. low). Stratified random sampling based on sex and age (< 75 years vs. ≥ 75 years) was applied by the public service of Ghent to select 1750 independently living older adults (≥ 65 years) from the 20 neighborhoods. Selected older adults were sent a letter with study information, and the notification of a home visit by a trained interviewer during the next 2 weeks. A maximum of three visit attempts were made to conduct the interview. In total, the trained interviewers found 1260 older adults at home, of which 633 agreed to participate (response rate: 50.2%). Of these, 125 (9.9%) were excluded due to severe physical restrictions, which resulted in a final sample of 508 older adults (response rate: 40.3%) [30].

#### Busschaert study

The public service of Sint-Niklaas (i.e. city in Flanders, Belgium) randomly selected 961 independently living older adults (≥ 65 years) from the municipal register [31]. Selected older adults received a letter with study information and the notification of a telephone call from a trained interviewer during the following days. A maximum of three telephone attempts were made to make an appointment for a home visit. The interviewers were able to reach 860 older adults by telephone, of which 293 agreed to participate (response rate: 30.5%). Of these, 35 older adults were excluded because they suffered from serious illness (*n* = 30), they did not speak Dutch (*n* = 4), or they were unable to stand up (*n* = 1). This resulted in a final sample of 258 older adults (response rate: 28.1%).

Consequently, a total of 766 older adults (508 from BEPAS Seniors and 258 from the Busschaert study) completed a structured face-to-face interview, took part in a grip strength test, and participated in body measurements. All measures were taken at home by trained researchers.

### Measures

#### Self-reported sedentary behaviors

Sedentary behaviors that were assessed in both the BEPAS Seniors and the Busschaert study included television time, computer time, motorized transport, reading, practicing hobbies (e.g. handicraft, playing cards), talking/listening to music, consuming meals, doing household activities, and making phone calls. The BEPAS Seniors questionnaire (see Additional file 1), developed by IDB and DVD and colleagues, asked the number of days a certain sedentary behavior was performed in the last 7 days, and the average time the participant engaged in that sedentary behavior on such a day. The average daily time spent in these sedentary behaviors was calculated using the following formula: (average number of days engaged in the behavior average * time engaged in the behavior on such a day) / 7. The Busschaert questionnaire, developed by IDB and colleagues, (see Additional file 2) asked how much time participants usually spent in one of the sedentary behaviors during the last 7 days on a weekday and on a weekend day [32]. The average daily time spent in sedentary behaviors was calculated by summing the weekday minutes (multiplied by five) and the weekend day minutes (multiplied by two), and by dividing the sum by seven. Test-retest reliability of items from both questionnaires was generally moderate to high, except for listening to music (ICC = 0.12), and practicing hobbies (ICC = 0.21) in the Busschaert study [33], and for household activities (ICC = 0.12) in the BEPAS Seniors [15]. Criterion validity of the questionnaires was moderate-to-good (ρ BEPAS questionnaire = 0.30 [34]; ρ Busschaert questionnaire = 0.48 [32]). To avoid the double report of simultaneous sedentary behaviors, participants were instructed to report only the main sedentary behavior (e.g., if one listens to the radio while reading a book, only reading was reported).

#### Physical and mental health outcomes

Physical and mental health items were assessed in the same way in both studies. Physical health outcomes included BMI, waist circumference, muscle strength, and physical health-related QOL. Mental health outcomes included mental health-related QOL. BMI was calculated based on body height and weight. Both body height and weight were measured to the nearest 0.1 cm and 0.1 kg, respectively, using a SECA portable stadiometer, and a weight scale. Waist circumference was measured three times with a flexible anthropometric tape at the level midway between the lower rib margin and the iliac crest with participants in standing position. The mean of the three measurements was taken as the final value. Upper body muscle strength was measured using a hand grip strength test. Participants were instructed to stand upright, and to hold the dynamometer in their dominant hand with the arm held out downwards (without making contact with the body). The test was executed twice, and a mean score was calculated. Physical and mental health-related QOL was estimated using the SF-12. The SF-12 is a widely used valid and reliable questionnaire that consists of 12 items measuring eight concepts relating to both mental and physical health-related QOL (i.e. physical functioning, role limitations caused by physical problems, bodily pain, general health, vitality, social functioning, role limitations caused by emotional problems and mental health) [35, 36]. A physical and a mental component score were calculated using item-specific weighted indicators and standardized from 0 to 100. Higher scores represent better functioning [37].

#### Socio-demographic characteristics

Sociodemographic characteristics included age, sex, family situation (having a partner; not having a partner), educational level (high (i.e., completed college or university); low (i.e., did not completed college or university)), and having children (yes; no). Detailed information on the included questions and answer categories can be found in Additional files 1 and 2.

### Statistical analysis

Before conducting the latent profile analyses, descriptive statistics were performed on socio-demographics, sedentary behaviors and health outcomes. Differences in socio-demographics, sedentary behaviors and health outcomes between men and women were analyzed using independent samples t-tests, chi^2^-tests, and Mann-Whitney U-tests, and the distribution of the sedentary behaviors was examined using SPSS 25. As household-related sitting time (both in men and women), and computer time (in women) were characterized by a large number of zeros (respectively, 73, 82 and 56%), these behaviors were excluded from further analyses. Moreover, sitting time when talking on the phone was also omitted from further analyses due to the limited time allocated to this behavior and the lack of variation (median = 2.14; Q1 = 0.0; Q3 = 7.5). As a result, seven sedentary behaviors were used to identify men’s sedentary behavior typologies: television time, computer time, transport-related sitting time, sitting for reading, sitting for hobbies, sitting for socializing and sitting for meals; and six sedentary behaviors were used to identify women’s sedentary behavior typologies: television time, transport-related sitting time, sitting for reading, sitting for hobbies, sitting for socializing and sitting for meals. Latent profile analyses were conducted in MPlus 8. The optimal number of typologies was determined based on a combination of fit criteria, typology sizes and the uniqueness of the typologies for each solution. Fit criteria included the sample-size adjusted Bayesian Information Criterion, the Bootstrap Likelihood Ratio Test, and the entropy values. Entropy values were expected to be above 80% in order to ensure that participants were assigned to the correct typology [38], and each typology was expected to represent at least 5% of the total sample [39]. Afterwards, the resulting typologies were imported in SPSS, and multivariate analyses of covariance were performed to assess differences in sedentary behaviors, and health-related outcome variables (i.e. BMI, waist circumference, grip strength, physical health-related QOL and the mental health-related QOL) between the sex-specific typologies, adjusting for age. Finally, chi^2^-tests and analyses of variances were executed to examine the association between socio-demographic characteristics and sex-specific typologies. *P*-values of less than 0.05 were considered statistically significant, and those below 0.10 were considered borderline significant.

## Results

### Sample characteristics

A total of 696 older adults were included in the current study. Socio-demographic, sedentary behavior and health-related characteristics of the sample are presented in Table 1 and in Additional file 3 (broken down by study sample). Briefly, participants had a mean age of 74.2 (SD = 6.2) years, ranging from 65.0 to 98.8 years. The majority of the participants had a partner, and children. About one third of the participants had a high educational level (i.e. college or university degree). Both in men and in women, highest levels of sedentary behavior were found for watching television, having meals, and reading. In women, mean BMI was 23.9 (SD = 4.9) kg/m^2^ and in men 25.4 (SD = 4.1) kg/m^2^. In total, 37.6% of the included women were overweight or obese, and 53.0% of the included men.

**Table 1:** Sample characteristics

	Total (*n* = 696)	Men (*n* = 323)	Women (*n* = 373)	Significance of difference
**Socio-demographic characteristics**				
Age: years, mean (SD)	74.2 (6.2)	73.7 (5.8)	74.6 (6.5)	T = 1.86, *p* = 0.07^a^
Family situation				
% having a partner	67.4%	81.1%	55.5%	X^**2**^ **= 51.12,** ***p*** **< 0.001**^**b**^
% having children	88.5%	88.2%	88.7%	X^2^ = 0.04, *p* = 0.83^b^
Educational level				
% with college/university degree	33.2%	36.4%	30.4%	X^2^ = 2.88, *p* = 0.09^b^
**Sedentary behaviors**				
Television time: min/day, median (Q1-Q3)	180.0 (90.0–240.0)	180.0 (90.0–240.0)	180.0 (90.0–240.0)	Z = -1.01, *p* = 0.31^c^
Computer time: min/day, median (Q1-Q3)	2.6 (0–60.0)	25.7 (0–90.0)	0 (0–34.3)	**Z = -5.36,** ***p*** **< 0.001**^**c**^
Transport-related sitting time: min/day, median (Q1-Q3)	22.5 (8.6–38.6)	25.7 (11.8–46.1)	21.4 (8.6–37.3)	**Z = -2.82,** ***p*** **= 0.01**^**c**^
Sitting for reading: min/day, median (Q1-Q3)	57.9 (28.9–90.0)	60.0 (30.0–90.0)	45.0 (22.5–90.0)	**Z = -2.34,** ***p*** **= 0.02**^**c**^
Sitting for hobbies: min/day, median (Q1-Q3)	5.4 (0–45)	0.0 (0–32.1)	16.1 (0–51.4)	**Z = -3.88,** ***p*** **< 0.001**^**c**^
Sitting for socializing: min/day, median (Q1-Q3)	30.0 (8.6–60.0)	30.0 (8.6–64.3)	30.0 (8.6–60.0)	Z = -0.50, *p* = 0.62^c^
Sitting for meals: min/day, median (Q1-Q3)	90 (60.0–90.0)	90.0 (60.0–90.0)	90 (90–90)	Z = -0.71, *p* = 0.48^c^
**Health-related outcomes**				
Body mass index: kg/m^2^, mean (SD)	24.3 (4.6)	25.4 (4.1)	23.9 (4.9)	**T = -4.17,** ***p*** **< 0.001**^**a**^
Waist circumference: cm, mean (SD)	95.6 (13.0)	101.1 (11.0)	90.9 (12.8)	**T = -10.91,** ***p*** **< 0.001**^**a**^
Grip strength: kg, mean (SD)	28.3 (10.8)	36.0 (9.9)	21.6 (6.1)	**T = -21.79,** ***p*** **< 0.001**^**a**^
Physical health-related QOL: mean (SD)	47.6 (9.1)	48.8 (8.5)	46.6 (9.5)	**T = -3.09,** ***p*** **= 0.002**^**a**^
Mental health-related QOL score: mean (SD)	49.1 (8.5)	50.0 (7.5)	48.2 (9.3)	**T = -2.82,** ***p*** **= 0.01**^**a**^

*SD* standard deviation, *Q1 – Q3* quartile 1 – quartile 3. The physical and mental health-related QOL were calculated with the scoring protocol of the SF12. Scores below 50 represent scores below the average in the population, whereas scores above 50 represent scores above the average in the population.^a^ = Independent Samples T-test, ^b^ = Chi-square test, ^c^ = Mann-Whitney U-test

### Sex-specific typologies of older adults’ sedentary behavior

Based on the sample-size adjusted Bayesian Information Criterium, the parametric Bootstrapped Likelihood Ratio Test, the Entropy and the class sizes, the 5-class model was selected as the optimal latent profile analysis solution for men (see Table 2).

**Table 2:** Model fit parameters for the two-, three-, four-, and five-class solution in men

Men: television time, computer time, transport-related sitting time, sitting for reading, sitting for hobbies, sitting for socializing and sitting for meals								
	Fit statistics			Profile Membership Distribution				
	SABIC	BLRT	Entropy	Profile 1	Profile 2	Profile 3	Profile 4	Profile 5
Two-profile	23,377.451	*P* < 0.001	0.96	0.11	0.89			
Three-profile	23,245.837	*P* < 0.001	0.94	0.11	0.78	0.11		
Four-profile	23,227.428	*P* < 0.001	0.87	0.58	0.10	0.21	0.11	
Five-profile	23,219.869	*P* < 0.001	0.83	0.22	0.45	0.14	0.11	0.9

*SABIC* sample-size adjusted Bayesian Information Criterium, *BLRT* the parametric Bootstrapped Likelihood Ratio Test

**Fig. 1:**
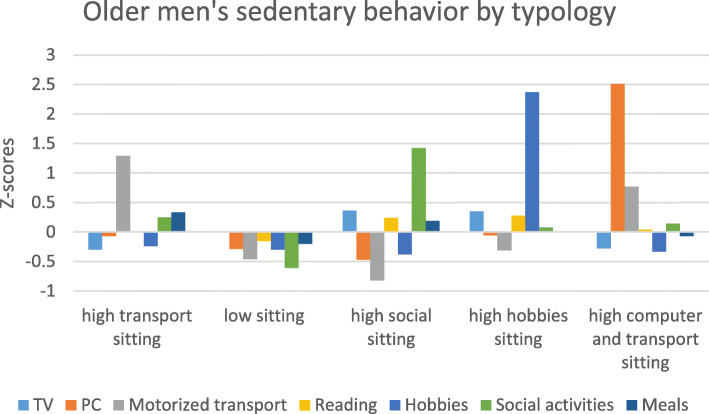
Standardized sedentary behaviors of older men by typology

The typologies for men are presented in Table 3 and Fig. 1. The first typology – named ‘high transport sitting’ – included 22.0% of the participants. The second typology ‘low sitting’ comprised almost half of the participants (44.6%). The third typology ‘high social sitting’ included 13.6% of the participants, and the fourth typology ‘high hobbies sitting’ included 11.1% of the participants. The smallest proportion of participants (8.7%) belongs to the fifth typology – termed ‘high computer and transport sitting’. All types of sedentary behavior differed between typologies, except sitting for reading. Significant differences are indicated in Table 3. Results of the pairwise comparisons are included in Additional file 4.

**Table 3:** Older men’s sedentary behavior by typology

	Typology 1 (22.0%) – high transport sitting	Typology 2 (44.6%) – low sitting	Typology 3 (13.6%) – high social sitting	Typology 4 (11.1%) – high hobbies sitting	Typology 5 (8.7%) – high computer and transport sitting	Significance of difference^
TV time (min/day)	147.15 (91.69)^c,d^	177.63 (98.70)	213.24 (96.06)^a^	211.90 (112.01)^a^	148.74 (88.71)	**F = 4.92,** ***p*** **= 0.001**
Computer time (min/day)	53.98 (53.52)^c,e^	35.72 (47.21)^e^	21.47 (42.36)^a,d,e^	55.22 (74.29)^c,e^	269.08 (45.51)^a,b,c,d^	**F = 129.54,** ***p*** **< 0.001**
Transport-related sitting time (min/day)	54.26 (7.77)^b,c,d,e^	19.57 (11.31)^a,c,e^	12.45 (10.24)^a,b,d,e^	22.42 (17.58)^a,c,e^	43.89 (19.87)^a,b,c,d^	**F = 127.62,** ***p*** **< 0.001**
Sitting for reading (min/day)	65.92 (43.64)	58.65 (44.60)	76.30 (48.05)	78.39 (42.96)	67.42 (48.44)	F = 2.22, *p* = 0.067
Sitting for hobbies (min/day)	13.61 (60.95)^d^	11.31 (17.86)^d^	8.23 (15.71)^d^	110.73 (30.12)^a,b,c,e^	9.95 (19.11)^d^	**F = 196.16,** ***p*** **< 0.001**
Sitting for socializing (min/day)	48.86 (33.66)^b,c^	18.73 (17.86) ^a,c,d,e^	89.63 (13.30) ^a,b,d,e^	42.79 (39.06)^b,c^	44.89 (32.11)^b,c^	**F = 66.98,** ***p*** **< 0.001**
Sitting for meals (min/day)	92.61 (32.88)^b^	75.13 (31.24)^a^	87.89 (29.79)	81.53 (32.60)	79.64 (36.49)	**F = 4.01,** ***p*** **= 0.003**
Total sitting time (min/day)	479.39 (131.49)^b,d,e^	396.75 (123.44)^a,c,d,e^	509.21 (124.43)^b,d,e^	602.98 (168.91)^a,b,c^	663.61 (123.56)^a,b,c^	**F = 36.64,** ***p*** **< 0.001**

^ Results of multivariate analysis of variance. Superscript letters and bold *p*-values represent significant differences between typologies. ^a^ significantly different from typology 1, ^b^ = significantly different from typology 2; ^c^ = significantly different from typology 3; ^d^ = significantly different from typology 4; ^e^ = significantly different from typology 5

The 3-class model was selected as the optimal latent profile analysis solution for women (see Table 4).

**Table 4:** Model fit parameters for the two-, three-, four-, and five-class solution in women

Women:: television time, transport-related sitting time, sitting for reading, sitting for hobbies, sitting for socializing and sitting for meals								
	Fit statistics			Profile membership distribution				
	SABIC	BLRT	Entropy	Profile 1	Profile 2	Profile 3	Profile 4	Profile 5
Two-profile	26,587.681	*P* < 0.001	0.98	0.92	0.08			
**Three-profile**	**26,524.772**	***P*** **< 0.001**	**0.88**	**0.69**	**0.08**	**0.24**		
Four-profile	26,378.155	*P* < 0.001	0.94	0.13	0.73	0.10	0.04	
Five-profile	26,383.319	*P* < 0.001	0.89	0.15	0.62	0.15	0.06	0.02

*SABIC* sample-size adjusted Bayesian Information Criterium, *BLRT* the parametric Bootstrapped Likelihood Ratio Test

**Fig. 2:**
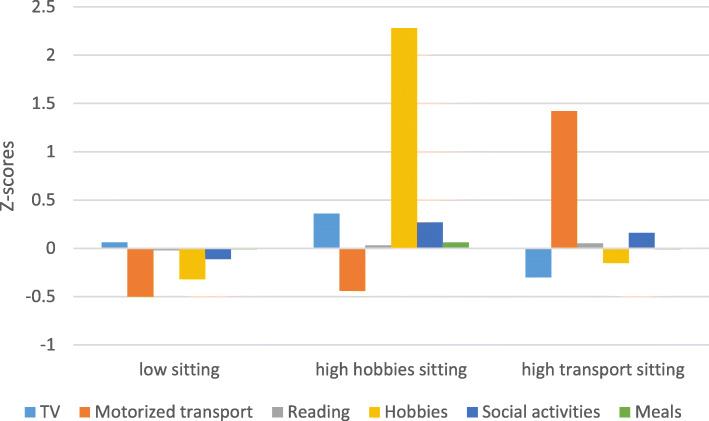
Standardized sedentary behaviors of older women by typology

The typologies for women are presented in Table 5 and Fig. 2. The first typology ‘low sitting’ represents the largest cluster with 63.5% of the participants. Typology 2 ‘high hobbies sitting’ is the smallest cluster with 10.7% of the participants. Typology 3 – named ‘high transport sitting’ comprises 25.7% of the participants. All types of sedentary behavior differed between typologies, except sitting for reading and sitting for meals. Significant differences are indicated in Table 5. Results of the pairwise comparisons are included in Additional file 4.

**Table 5:** Older women’s sedentary behavior by typology

	Typology 1 (63.5%) – low sitting	Typology 2 (10.7%) – high hobbies sitting	Typology 3 (25.7%) – high transport sitting	Significance of difference^
TV time (min/day)	191.8 (101.45)^c^	222.6 (105.7)^c^	154.7 (95.1)^a,b^	**F = 7.72,** ***p*** **= 0.001**
Transport-related sitting time (min/day)	15.0 (10.0)^c^	16.2 (11.5)^c^	50.4 (9.5)^a,b^	**F = 438.16,** ***p*** **< 0.001**
Sitting for reading (min/day)	57.2 (44.3)	59.5 (52.5)	60.4 (42.6)	F = 0.20, *p* = 0.823
Sitting for hobbies (min/day)	17.8 (21.9)^b^	126.0 (32.5)^a,c^	24.8 (29.9)^b^	**F = 314.21,** ***p*** **< 0.001**
Sitting for socializing (min/day)	34.0 (31.9)	46.7 (39.1)	43.1 (31.7)	**F = 4.36,** ***P*** **= 0.013**
Sitting for meals (min/day)	80.1 (31.8)	82.2 (39.9)	80.0 (29.4)	F = 0.08, *p* = 0.923
Total sitting time (min/day)	395.84 (122.67)^b^	553.29 (135.34)^a,c^	413.33 (112.84)^b^	F = 28.73, *p* < 0.001

^ Results of multivariate analysis of variance. Superscript letters and bold p-values represent significant differences between typologies. ^a^ significantly different from typology 1, ^b^ = significantly different from typology 2; ^c^ = significantly different from typology 3; ^d^ = significantly different from typology 4; ^e^ = significantly different from typology 5

### Differences in health-related outcomes and socio-demographic characteristics between typologies in men

No overall significant differences in health-related outcomes were found between the five identified typologies in men (see Table 6). However, pairwise comparisons showed that men that are highly engaged in sedentary hobbies (i.e. typology 4) had a significant lower score on physical health-related QOL than men that are highly engaged in motorized transport (i.e. typology 1) (*p* = 0.01). Borderline significant differences were found in BMI, grip strength, and mental health-related QOL between typologies. Concretely, men that are highly engaged in sedentary social activities (i.e. typology 3) had a higher BMI than men characterized by relatively low levels of overall sedentary behavior (i.e. typology 2) (*p* = 0.08). Men that are highly engaged in motorized transport (i.e. typology 1) and men that are highly engaged in motorized transport and computer use (i.e. typology 5) had a higher grip strength than men that are highly engaged in sedentary hobbies (i.e. typology 4) (both *p* = 0.06). They had also a higher score on mental health-related QOL than men characterized by relatively low levels of overall sedentary behavior (i.e. typology 2) (respectively, *p* = 0.08 and *p* = 0.09). Detailed results of the pairwise comparisons are included in Additional file 5.

**Table 6:** Differences in health-related outcomes and socio-demographic characteristics by typology (men)

	Typology 1 (22.0%) – high transport sitting	Typology 2 (44.6%) – low sitting	Typology 3 (13.6%) – high social sitting	Typology 4 (11.1%) – high hobbies sitting	Typology 5 (8.7%) – high computer and transport sitting	Significance of difference^
**Health-related outcomes**						
BMI (kg/m^2^)	25.2 (4.0)	25.0 (4.2)^c^	26.3 (4.5)^b^	26.0 (4.4)	25.3 (3.3)	F = 1.10, *p* = 0.358
Waist circumference (cm)	100.7 (10.4)	100.9 (11.1)	102.0 (11.2)	100.3 (12.1)	102.7 (11.4)	F = 0.25, *p* = 0.909
Grip strength (kg)	38.6 (9.4)^d^	35.8 (10.2)	33.5 (10.4)	33.8 (7.9)^a,e^	40.4 (9.3)^d^	F = 1.52, *p* = 0.196
Physical health-related QOL	51.2 (8.2)^d^	48.8 (8.4)	48.2 (8.0)	46.0 (8.3)^a^	48.9 (9.9)	F = 1.80, *p* = 0.129
Mental health-related QOL	51.2 (8.3)^b^	48.9 (7.9)^a,e^	50.0 (6.3)	50.8 (4.8)	52.1 (7.5)^b^	F = 1.30, *p* = 0.270
**Socio-demographic characteristics**						
Age: years, mean (SD)	72.5 (5.2)^c^	74.3 (6.3)^e^	76.1 (5.5)^a,e^	73.8 (5.3)	70.4 (3.8)^b,c^	**F = 5.33, p < 0.001**
Family situation						
% having a partner	87.3%	79.2%	74.4%	75.0%	92.9%	X^2^ = 6.78, *p* = 0.15
% having children	91.5%	84.7%	83.7%	91.7%	100.0%	X^2^ = 7.43, p = 0.12
Educational level						
% with college/university degree	14.1%	10.6%	7.1%	27.8%	17.9%	X^**2**^ **= 15.69, p = 0.04**

^ Results of multivariate analysis of (co)variance (adjusted for age), and chi-square tests. Superscript letters and bold *p*-values represent (borderline) significant differences between typologies. ^a^ significantly different from typology 1, ^b^ = significantly different from typology 2; ^c^ = significantly different from typology 3; ^d^ = significantly different from typology 4; ^e^ = significantly different from typology 5

Two overall significant differences in socio-demographic characteristics were found between the five identified typologies in men (see Table 6); specifically for age and educational level. Men that are highly engaged in sedentary social activities (i.e. typology 3) were more likely to be older, and men that are highly engaged in motorized transport and computer use (i.e. typology 5) were more likely to be younger. Men characterized by relatively low levels of overall sedentary behavior (i.e. typology 2), and men that are highly engaged in sedentary social activities (i.e. typology 3) were more likely to have completed college or university, and men that are highly engaged in sedentary hobbies were more likely to have not completed college or university.

### Differences in health-related outcomes and socio-demographic characteristics between typologies in women

A (borderline) significant difference was found in BMI, waist circumference and physical health-related QOL between the identified typologies in women (see Table 7). Pairwise comparison showed that women that are highly engaged in sedentary hobbies (i.e. typology 2) had a significantly higher BMI than women characterized by low levels of overall sedentary behavior (i.e. typology 1) (*p* = 0.002) and women that are highly engaged in motorized transport (i.e. typology 3) (*p* = 0.01). Women that are highly engaged in sedentary hobbies (i.e. typology 2) also had a higher waist circumference than women characterized by low levels of overall sedentary behavior (i.e. typology 1) (*p* = 0.02). Women that are highly engaged in motorized transport (i.e. typology 3) scored significantly higher on physical health-related QOL compared to women characterized by low levels of overall sedentary behavior (i.e. typology 1) (*p* = 0.04) and women that are highly engaged in sedentary hobbies (i.e. typology 2) (*p* = 0.02). Pairwise comparisons also showed a borderline significant difference in grip strength, and mental health-related QOL. Specifically, women that are highly engaged in motorized transport (i.e. typology 3) had a higher grip strength than women characterized by low levels of overall sedentary behavior (i.e. typology 1) (*p* = 0.07) and women that are highly engaged in sedentary hobbies (i.e. typology 2) (*p* = 0.09); they had also a higher score on mental health-related QOL than women that are highly engaged in sedentary hobbies (i.e. typology 2) (*p* = 0.09).

**Table 7:** Differences in health-related outcomes by typology (women)

	Typology 1 (63.5%) – low sitting	Typology 2 (10.7%) – high hobbies sitting	Typology 3 (25.7%) – high transport sitting	Significance of difference^
**Health-related outcomes**				
BMI (kg/m^2^)	23.5 (5.1)^b^	26.3 (4.0)^a,c^	23.4 (4.3)^b^	**F = 5.22,** ***p*** **= 0.006**
Waist circumference (cm)	89.9 (12.7)^b^	95.3 (14.5)^a^	90.5 (12.2)	**F = 2.71,** ***p*** **= 0.068**
Grip strength (kg)	21.3 (6.2)^c^	20.7 (4.4)^c^	23.4 (6.3)^a,b^	F = 2.15, *p* = 0.118
Physical health-related QOL	46.1 (9.9)^c^	44.1 (8.3)^c^	49.3 (8.3)^a,b^	**F = 3.54,** ***p*** **= 0.030**
Mental health-related QOL	48.0 (9.5)	46.2 (8.9)^c^	49.1 (8.7)^b^	F = 1.50, *p* = 0.225
**Socio-demographic characteristics**				
Age: years, mean (SD)	75.1 (6.7)^c^	75.1 (6.1)	73.1 (5.9)^a^	**F = 3.67,** ***p*** **= 0.027**
Family situation				
% having a partner	55.9%	51.3%	56.2%	X^2^ = 0.32, *p* = 0.85
% having children	89.9%	89.7%	85.4%	X^2^ = 1.40, *p* = 0.50
Educational level				
% with college/university degree	22.9%	11.4%	16.5%	X^2^ = 3.48, *p* = 0.18

^ Results of multivariate analysis of (co)variance (adjusted for age), and chi-square tests. Superscript letters and bold *p*-values represent (borderline) significant differences between typologies. ^a^ significantly different from typology 1, ^b^ = significantly different from typology 2; ^c^ = significantly different from typology 3

With regard to socio-demographic characteristics, an overall significant differences was found in age between the three identified typologies in women (see Table 7); specifically women characterized by low levels of overall sedentary behavior (i.e. typology 1) were more likely to be older than women that are highly engaged in motorized transport (i.e. typology 3) (*p* = 0.02).

## Discussion

This study examined sex-specific typologies of sedentary behaviors and their cross-sectional associations with health-related outcomes and socio-demographic characteristics in older adults. To the best of our knowledge, no previous studies have identified sex-specific sedentary behavioral typologies in older adults. Nevertheless, previous studies have suggested that not all sedentary behaviors may be similarly associated with physical and mental health risks in older adults, and thus, understanding the associations between sedentary behavioral typologies and health-related outcomes is important to inform risk stratification and preventive interventions. Insight into the socio-demographic differences between typologies is useful to target at-risk populations.

Results of the latent profile analyses identified five unique typologies in men, and three in women. Typologies differed most on computer time, motorized transport and sedentary hobbies, and least on meals and reading. The majority of the typologies had at least one dominating sedentary behavior that distinguishes it from the other typologies. Only the most common typology – i.e. the one characterized by low overall levels of sedentary behavior – had no clear dominating sedentary behavior in both sexes. Although the latter typology is labelled ‘low sitting’, it should be noted that this is based on relative values and that older adults of this typology are still spending most of their time sedentary. When analyzing the typologies in detail, it becomes clear that older adults’ motorized transport and computer time tend to cluster (in men), and that television time is generally opposite to computer time (in men) and to motorized transport (in both sexes). The opposition between television time and computer time is not unexpected since different correlates are identified for both types of sedentary behavior [15, 40, 41]. For example, lower educated older adults have been shown to be more likely to watch television, whereas higher educated adults have been shown to be more likely to use the computer. The latter finding is supported by the results of our analyses with socio-demographic characteristics. The coexistence of older adults’ computer time and motorized transport, on the other hand, was less expected and has, to our knowledge, not been identified in the literature. More research is needed to confirm this finding, and to examine if older adults’ computer time and motorized transport share the same correlates.

Results of the analyses of covariance showed that certain typologies of sedentary behaviors are indeed more strongly related to negative health outcomes than others. Unfortunately, the cross-sectional nature of this study prevents drawing causal inferences from the associations. Participants of typologies with high motorized transport and/or computer time (i.e. typology 1 and 5 in men, and typology 3 in women) generally have better health outcomes; i.e. they scored better on the grip strength test, and had a better physical and mental health-related QOL compared to participants of other typologies. Or vice versa, participants of typologies with high television time (i.e. typology 3 and 4 in men, and typology 2 in women) scored less well on health-related outcomes, like BMI, grip strength, physical health-related QOL, and mental health-related QOL. Although some of the differences between typologies in health-related outcomes are not statistically significant, they can be considered clinically relevant based on the results of recent observational studies [42, 43] and meta-analyses [44, 45]. For example, a difference of 7 kg in men’s grip strength is important, as a large-scale prospective cohort study revealed a hazard ratio for all-cause mortality of 1.16 for each 5 kg lower grip strength [42]. Similarly, a difference of 6 cm in women’s waist circumference is relevant, as a meta-analysis revealed a hazard ratio for obesity-related cancers of 1.13 per standard deviation increment in waist circumference [45].

The fact that typologies with high television time scored less well on health-related outcomes is in line with previous studies showing that television time is strongly associated with cardiovascular diseases, metabolic syndrome, and all-cause mortality [22, 46]. Although the underlying mechanisms for the stronger relationships between television time and negative health outcomes are still not fully understood, it can be assumed that the associated unhealthy dietary habits play an important role [26, 47]. Next to reducing television time, it can also be recommended to focus on the increase of (at least moderate intensity) physical activity, as the increased health risks pertaining to prolonged television time (and sitting time in general) appear to be attenuated by increased amounts of moderate-to-vigorous intensity physical activity [48, 49].

In contrast to our expectations, participants belonging to the typology represented by relatively low levels of overall sedentary behavior (i.e. typology 2 in men, and typology 1 in women) were not the ones with the most positive (physical) health outcomes. Although they have a lower BMI (both in men and in women), and a lower waist circumference (in women), they did not score better, or even worse, on grip strength, physical health-related QOL, and mental health-related QOL compared to participants of typologies represented by high motorized transport (i.e. typology 1 and 5 in men, and 3 in women). The positive associations between motorized transport and health outcomes are in line with previous studies [50, 51], and are assumed to be bidirectional. Older adults with physical health problems, impaired mobility, and visual and cognitive deficit might experience difficulties to drive a car, as car driving is a complex activity requiring a range of cognitive and psychomotor abilities [52]. These difficulties can make them reduce, or even cease, driving a car [53]. On the other hand, participants who do not drive a car might experience transportation deficiency [54] and face social exclusion [55], which might affect older adults’ mental health. Given that social interaction as well as engagement in social activities are basic components of successful aging [50], it is recommended that healthy aging researchers focus on older adults who are in the transition to driving cessation, and on the increase of alternative transport modes, such as public transport and e-bikes [56], rather than on reducing transport-related sitting time.

A major strength of this study is its uniqueness, as no previous studies have identified typologies of older adults’ sedentary behaviors, and have linked these typologies with health outcomes, and socio-demographic characteristics. A second strength is the use of objective measures (BMI, waist circumference, and grip strength), which were assessed using standardized examinations. A third strength is the application of face-to-face interviews to complete the validated questionnaires (sedentary behavior and health-related QOL). The use of face-to-face interviews is recommended in older adults, as some older adults may experience cognitive difficulties when responding to paper-based questionnaires [57]. Important limitations of the current study are its cross-sectional design, which does not allow establishing causal relationships. Although there is good evidence for the causal influence of sedentary behaviors on weight status [58] and health-related QOL [59], these health outcomes may also causally influence sedentary behaviors. A second limitation is the low response rate, which raises the probability of response bias. While all socio-demographic subgroups are well represented in the sample, it remains plausible that participants who are more concerned with their health are overrepresented. Finally, the study is limited by the lack of information on cognitive functioning, cognitive impairment and social health. Cognitive decline and impairment, and social health problems have been shown to be highly prevalent in older adults, and are serious threats to older adults’ independence, quality of life, and daily life functional abilities [60]. As some types of sedentary behavior might be protective for cognitive decline, and social exclusion [24], future studies should include cognitive functioning and social health measures.

## Conclusion

In conclusion, five different sedentary behavioral typologies were identified in older men, and three in older women. As expected, identified sedentary behavioral typologies were not equally related to physical and mental health outcomes. Consistent with the broader literature, typologies that are characterized by high television time seem to be related to more negative health outcomes, like a higher BMI, less grip strength, and a lower physical and mental health-related QOL. Typologies that are represented by high computer time and motorized transport, on the other hand, seem to be related to more positive health outcomes, such as a lower BMI, more grip strength and a higher physical and mental health-related QOL. Although causal direction between identified typologies and health outcomes cannot be determined due to the cross-sectional nature of the study, our results suggests that future interventions should better focus on specific types of sedentary behavior (e.g. television time), or patterns of sedentary behavior, and not on total sedentary behavior. Reducing transport-related sitting time might not be recommended, as this type of sedentary behavior seems to contribute to healthy aging. Future research using longitudinal designs is required to further unravel the causal mechanisms underlying the detected relationships, and to optimally inform the development of public health interventions.

## Supplementary Information

**Additional file 1.** English language translation of the BEPAS Seniors questionnaire

**Additional file 2.** English language translation of the Busschaert questionnaire

**Additional file 3.** Descriptive statistics broken down by study

**Additional file 4.** Older adults’ sedentary behaviors by typology - results of the pairwise comparisons

**Additional file 5.** Differences in health-related outcomes and socio-demographics depending on typology – results of the pairwise compairsons

## Data Availability

The datasets used and/or analyzed during the current study are available from the corresponding author on reasonable request.

## Abbreviations

BMI: Body Mass Index
QOL: Quality-of-life
BEPAS Seniors: Belgian Environmental Physical Activity Study in Seniors
