# Supplementary material for: Sex-specific typologies of older adults’ sedentary behaviors and their associations with health-related and socio-demographic factors: a latent profile analysis
Source: BMC Geriatr. 2021 Jan 19;21:66. doi: 10.1186/s12877-021-02011-5 (PMC7816402; doi:10.1186/s12877-021-02011-5)
Supplement: Supplementary file 1 — Additional file 1. English language translation of the BEPAS Seniors questionnaire [file 12877_2021_2011_MOESM1_ESM.pdf]

BEPAS seniors questionnaire

We would like to find out more information about the way you think about your health.

1. GENERAL HEALTH:

In general, would you say your health is:

O Excellent

O Very Good

O Good

O Fair

O Poor

Compared to one year ago, how would you rate your health in general now?

O Much better now than one year ago

O Somewhat better now than one year ago

O About the same

O Somewhat worse now than one year ago

O Much worse than one year ago

Limitations of activities:

The following items are about activities you might do during a typical day. Does your health now limit you in these activities? If so, how much?

Vigorous activities, such as running, lifting heavy objects, participating in strenuous sports.

O Yes, Limited a lot

O Yes, Limited a Little

O No, Not Limited at all

Moderate activities, such as moving a table, pushing a vacuum cleaner, bowling, or playing golf

O Yes, Limited a lot

O Yes, Limited a Little

O No, Not Limited at all

Lifting or carrying groceries

O Yes, Limited a lot

O Yes, Limited a Little

O No, Not Limited at all

Climbing several flights of stairs

O Yes, Limited a lot

O Yes, Limited a Little

O No, Not Limited at all

Climbing one flight of stairs

O Yes, Limited a lot

O Yes, Limited a Little

O No, Not Limited at all

Bending, kneeling, or stooping

O Yes, Limited a lot

O Yes, Limited a Little

O No, Not Limited at all

Walking more than a mile

O Yes, Limited a lot

O Yes, Limited a Little

O No, Not Limited at all

Walking several blocks

O Yes, Limited a lot

O Yes, Limited a Little

O No, Not Limited at all

Walking one block

O Yes, Limited a lot

O Yes, Limited a Little

O No, Not Limited at all

Bathing or dressing yourself

O Yes, Limited a lot

O Yes, Limited a Little

O No, Not Limited at all

Physical health problems:

During the past 4 weeks, have you had any of the following problems with your work or other regular daily activities as a result of your physical health?

Cut down the amount of time you spent on work or other activities

O Yes

O No

Accomplished less than you would like

O Yes

O No

Were limited in the kind of work or other activities

O Yes

O No

Had difficulty performing the work or other activities (for example, it took extra effort)

O Yes

O No

Emotional health problems:

During the past 4 weeks, have you had any of the following problems with your work or other regular daily activities as a result of any emotional problems (such as feeling depressed or anxious)?

Cut down the amount of time you spent on work or other activities

O Yes

O No

Accomplished less than you would like

O Yes

O No

Didn't do work or other activities as carefully as usual

O Yes

O No

Social activities:

Emotional problems interfered with your normal social activities with family, friends, neighbors, or groups?

O Not at all

O Slightly

O Moderately

O Severe

O Very Severe

Pain:

How much bodily pain have you had during the past 4 weeks?

O None

O Very Mild

O Mild

O Moderate

O Severe

O Very Severe

During the past 4 weeks, how much did pain interfere with your normal work (including both work outside the home and housework)?

O Not at all

O A little bit

O Moderately

O Quite a bit

O Extremely

Energy and emotions:

These questions are about how you feel and how things have been with you during the last 4 weeks. For each question, please give the answer that comes closest to the way you have been feeling.

Did you feel full of pep?

O All of the time

O Most of the time

O A good Bit of the Time

O Some of the time

O A little bit of the time

O None of the Time

Have you been a very nervous person?

O All of the time

O Most of the time

O A good Bit of the Time

O Some of the time

O A little bit of the time

O None of the Time

Have you felt so down in the dumps that nothing could cheer you up?

O All of the time

O Most of the time

O A good Bit of the Time

O Some of the time

O A little bit of the time

O None of the Time

Have you felt calm and peaceful?

O All of the time

O Most of the time

O A good Bit of the Time

O Some of the time

O A little bit of the time

O None of the Time

Did you have a lot of energy?

O All of the time

O Most of the time

O A good Bit of the Time

O Some of the time

O A little bit of the time

O None of the Time

Have you felt downhearted and blue?

O All of the time

O Most of the time

O A good Bit of the Time

O Some of the time

O A little bit of the time

O None of the Time

Did you feel worn out?

O All of the time

O Most of the time

O A good Bit of the Time

O Some of the time

O A little bit of the time

O None of the Time

Have you been a happy person?

O All of the time

O Most of the time

O A good Bit of the Time

O Some of the time

O A little bit of the time

O None of the Time

Did you feel tired?

O All of the time

O Most of the time

O A good Bit of the Time

O Some of the time

O A little bit of the time

O None of the Time

Social activities:

During the past 4 weeks, how much of the time has your physical health or emotional problems interfered with your social activities (like visiting with friends, relatives, etc.)?

O All of the time

O Most of the time

O A good Bit of the Time

O Some of the time

O A little bit of the time

O None of the Time

General health:

How true or false is each of the following statements for you?

I seem to get sick a little easier than other people

O Definitely true

O Mostly true

O Don't know

O Mostly false

O Definitely false

I am as healthy as anybody I know

O Definitely true

O Mostly true

O Don't know

O Mostly false

O Definitely false

I expect my health to get worse

O Definitely true

O Mostly true

O Don't know

O Mostly false

O Definitely false

My health is excellent

O Definitely true

O Mostly true

O Don't know

O Mostly false

O Definitely false

2. PHYSICAL ACTIVITY:

I am going to ask you about the time you spent being physically active in the last 7 days. Please answer each question even if you do not consider yourself to be an active person. Think about the activities you do at work, as part of your house and yard work, to get from place to place, and in your spare time for recreation, exercise or sport.

PART 1: JOB-RELATED PHYSICAL ACTIVITY

READ: The first questions are about your work. This includes paid jobs, farming, volunteer work, course work and any other unpaid work that you did outside your home. Do not include unpaid work you might do around your home, like housework, yard work, general maintenance, and caring for your family. I will ask you about these later.

Do you currently have a job or do any unpaid work outside your home?

_____ Yes

_____ No

[**Interviewer clarification**: This also includes credit and non-credit classes or course work. It also includes volunteer work and time spent looking for work. It does not includes unpaid house or yard work, nor caring for dependents, this will be asked in a later section.]

The following questions are about all the physical activity you did as part of your paid or unpaid work. This does not include traveling to and from work.

First, think about all the *moderate-to-vigorous* activities that you did as part of your work.
<u>Vigorous activities</u> make you breathe much harder than normal. These may include things like heavy lifting, digging, heavy construction work, or climbing up stairs. Think about only those vigorous physical activities that you did for at least 10 minutes at a time.
<u>Moderate physical activities</u> make you breathe somewhat harder than normal and may include activities like carrying light loads. Do not include walking. Again, think about only those moderate physical activities that you did for at least 10 minutes at a time.

During the **last 7 days**, on how many days did you do **moderate-to-vigorous** physical activities **as part of your work**?

_____ Days per week

[**Interviewer clarification**: Think about only those physical activities that you did for at least 10 minutes at a time.]

[**Interviewer clarification**: Work includes paid and unpaid work as well as course work. Include all jobs and volunteer work.

How much time did you usually spend on one of those days doing **moderate-to-vigorous** physical activities as part of your work?

___ ___ Hours per day
___ ___ Minutes per day

[**Interviewer clarification**: Think about only those physical activities you did for at least 10 minutes at a time.]

[**Interviewer probe**: An average time per day is being sought. If the respondent can't answer because the pattern of time spent varies widely from day to day, or includes time spent doing a variety of paid and unpaid work, ask: "What is the total amount of time you spent **over the last 7 days** doing vigorous physical activities as part of your work?"

___ ___ Hours per week
___ ___ ___ ___ Minutes per week

Now think about the time you spend *walking* for at least 10 minutes at a time as part of your work. Please do not count any walking you did to travel to or from work.

During the **last 7 days**, on how many days did you **walk as part of your work**?

_____ Days per week

[**Interviewer clarification**: Think about only the walking that you did for at least 10 minutes at a time.]

[**Interviewer clarification**: Include all jobs.]

How much time did you usually spend on one of those days **walking** as part of your work?

___ ___ Hours per day
___ ___ ___ Minutes per day

[**Interviewer clarification**: Think about only the walking you did for at least 10 minutes at a time.]

If you have walked as part of your work during the last 7 days, how would you describe your walking speed?

O High
O Moderate
O Low

PART 2: TRANSPORTATION PHYSICAL ACTIVITY

READ: Now, think about how you traveled from place to place, including to places like work, stores, movies and so on.

During the **last 7 days**, on how many days did you **travel in a motor vehicle** like a train, bus, car or tram?

_____ Days per week

How much time did you usually spend on one of those days **traveling** in a car, bus, train or other kind of motor vehicle?

___ ___ Hours per day
___ ___ ___ Minutes per day

[**Interviewer probe**: An average time per day is being sought. If the respondent can't answer because the pattern of time spent varies widely from day to day, ask: "What is the total amount of time you spent **over the last 7 days** traveling in a motor vehicle?"

___ ___ Hours per week
___ ___ ___ ___ Minutes per week

Now think only about the *bicycling* you did to travel to and from work, to do errands, or to go from place to place. Only include bicycling that you did for at least 10 minutes at a time.

During the **last 7 days**, on how many days did you **bicycle** to go from place to place?

_____ Days per week

[**Interviewer clarification**: Think only about the bicycling that you did for at least 10 minutes at a time.]

How much time did you usually spend on one of those days **to bicycle** from place to place?

___ ___ Hours per day
___ ___ ___ Minutes per day

[**Interviewer clarification**: Think about only the bicycling that you did for at least 10 minutes at a time.]

[**Interviewer probe**: An average time per day is being sought. If the respondent can't answer because the pattern of time spent varies widely from day to day, ask: "What is the total amount of time you spent bicycling **over the last 7 days** to travel from place to place?"

___ ___ Hours per week

___ ___ ___ ___ Minutes per week

Now think only about the *walking* you did to travel to and from work, to do errands or to go from place to place. Only include walking that you did for at least 10 minutes at a time.

During the **last 7 days**, on how many days did you **walk to go from place to place**?

_____ Days per week

[**Interviewer clarification**: Think only about the walking that you did for at least 10 minutes at a time.]

How much time did you usually spend on one of those days **walking from place to place**?

___ ___ Hours per day

___ ___ ___ Minutes per day

[**Interviewer clarification**: Think about only the walking that you did for at least 10 minutes at a time.]

If you have walked to go from place to place during the last 7 days, how would you describe your walking speed?

O High
O Moderate
O Low

PART 3: HOUSEWORK, HOUSE MAINTENANCE AND CARING FOR FAMILY

Now think about the physical activities you have done in the last 7 days in and around your home, like housework, gardening, yard work, general maintenance work, and caring for your family.

First think about *moderate-to-vigorous* activities that you did in the garden or yard.

<u>Vigorous activities</u> **make you breathe much harder than normal. These may include things like heavy lifting, digging, heavy construction work, or climbing up stairs. Think about only those vigorous physical activities that you did for at least 10 minutes at a time.**

<u>Moderate physical activities</u> **make you breathe somewhat harder than normal and may include activities like carrying light loads. Do not include walking. Again, think about only those moderate physical activities that you did for at least 10 minutes at a time.**

During the **last 7 days**, on how many days did you do **moderate-to-vigorous** physical activities **in the garden or yard**?

_____ Days per week

[**Interviewer clarification**: Think about only those physical activities that you did for at least 10 minutes at a time.]

How much time did you usually spend on one of those days doing **moderate-to-vigorous** physical activities in the garden or yard?

___ ___ Hours per day

___ ___ ___ Minutes per day

[**Interviewer clarification**: Think about only those physical activities that you did for at least 10 minutes at a time.]

Now think about activities which take at least *moderate* physical effort that you did *inside your home*. Examples include carrying light loads, washing windows, scrubbing floors, and sweeping. Include only those moderate physical activities that you did for at least 10 minutes at a time.

[**Interviewer clarification**: Moderate activities make you breathe somewhat harder than normal.]

During the **last 7 days**, on how many days did you do **moderate** activities **inside your home**?

_____ Days per week

[**Interviewer clarification**: Think about only those physical activities that you did for at least 10 minutes at a time.]

[**Interviewer clarification**: During the last 7 days, on how many days did you do activities that take **at least moderate** effort inside your home?]

How much time did you usually spend on one of those days doing **moderate** physical activities inside your home?

___ ___ Hours per day

___ ___ ___ Minutes per day

[**Interviewer clarification**: Think about only those physical activities that you did for at least 10 minutes at a time.]

PART 4: RECREATION, SPORT, AND LEISURE-TIME PHYSICAL ACTIVIITY

Now, think about all the physical activities that you did in the last 7 days solely for recreation, sport, exercise or leisure. Please do not include any activities you have already mentioned.

Not counting any cycling you have already mentioned, during the **last 7 days**, on how many days did you **cycle** for at least 10 minutes at a time **in your leisure time**?

_____ Days per week

[**Interviewer clarification**: Think about only the cycling that you did for at least 10 minutes at a time.]

How much time did you usually spend on one of those days **cycling** in your leisure time?

___ ___ Hours per day

___ ___ ___ Minutes per day

[**Interviewer clarification**: Think about only the walking that you did for at least 10 minutes at a time.]

Not counting any walking you have already mentioned, during the **last 7 days**, on how many days did you **walk** for at least 10 minutes at a time **in your leisure time**?

_____ Days per week

[**Interviewer clarification**: Think about only the walking that you did for at least 10 minutes at a time.]

How much time did you usually spend on one of those days **walking** in your leisure time?

___ ___ Hours per day

___ ___ ___ Minutes per day

[**Interviewer clarification**: Think about only the walking that you did for at least 10 minutes at a time.]

If you have walked in your leisure time during the last 7 days, how would you describe your walking speed?

O High

O Moderate

O Low

Now think about other physical activities you did in your leisure time for at least 10 minutes at a time.

First, think about *moderate-to-vigorous* activities that you did in your leisure time. Examples include aerobics, running, fast bicycling, or fast swimming.

During the **last 7 days**, on how many days did you do **moderate-to-vigorous** physical activities **in your leisure time**?

_____ Days per week

[**Interviewer clarification**: Think about only those moderate-to-vigorous physical activities that you did for at least 10 minutes at a time.]

How much time did you usually spend on one of those days doing **moderate-to-vigorous** physical activities in your leisure time?

___ ___ Hours per day

___ ___ ___ Minutes per day

[**Interviewer clarification**: Think about only those physical activities that you did for at least 10 minutes at a time.]

PART 5: TIME SPENT SITTING

READ: The last question is about the time that you spent *sitting* during the last 7 days. Include time at work, at home, while doing course work and during leisure time. This may include time spent sitting at a desk, visiting friends, reading or sitting or lying down to watch television. Do also include any time spent sitting in a motor vehicle that you have already told me about.

During the **last 7 days**, on how many days did you travel by public transport, including the train, bus or tram?

_____ Days per week

How much time did you usually spend **sitting** while travelling by public transport on one of those days?

___ ___ Hours per day

___ ___ ___ Minutes per day

During the **last 7 days**, on how many days did you travel by a motorized vehicle, such as car, motorbike or moped, of which you were the driver?

_____ Days per week

How much time did you usually spend **sitting** while travelling by a motorized vehicle of which you were the driver on one of those days?

___ ___ Hours per day

___ ___ ___ Minutes per day

During the **last 7 days**, on how many days did you travel by a motorized vehicle, such as car, motorbike or moped, of which you were not the driver?

_____ Days per week

How much time did you usually spend **sitting** while travelling by a motorized vehicle of which you were not the driver on one of those days?

___ ___ Hours per day

___ ___ ___ Minutes per day

During the **last 7 days**, on how many days did you use a computer?

_____ Days per week

How much time did you usually spend **sitting** while using a computer on one of those days?

___ ___ Hours per day

___ ___ ___ Minutes per day

During the **last 7 days**, on how many days did you read a book, a journal or a newspaper?

_____ Days per week

How much time did you usually spend **sitting** while reading a book, journal or newspaper on one of those days?

___ ___ Hours per day

___ ___ ___ Minutes per day

During the **last 7 days**, on how many days did you watch television?

_____ Days per week

How much time did you usually spend **sitting** while watching television on one of those days?

___ ___ Hours per day

___ ___ ___ Minutes per day

During the **last 7 days**, on how many days did perform other sitting hobbies (e.g. handcraft, playing cards etc.)?

_____ Days per week

How much time did you usually spend **sitting** while performing these other hobbies on one of those days?

___ ___ Hours per day

___ ___ ___ Minutes per day

During the **last 7 days**, on how many days did you make a phone call?

_____ Days per week

How much time did you usually spend **sitting** while making a phone call on one of those days?

___ ___ Hours per day

___ ___ ___ Minutes per day

During the **last 7 days**, on how many days did you talk to friends or family, or did you listen to music?

_____ Days per week

How much time did you usually spend **sitting** while talking to friend/family or while listing to music on one of those days?

___ ___ Hours per day

___ ___ ___ Minutes per day

During the **last 7 days**, on how many days did you perform household tasks?

_____ Days per week

How much time did you usually spend **sitting** while performing household tasks on one of those days?

___ ___ Hours per day

___ ___ ___ Minutes per day

During the **last 7 days**, on how many days did you consume meals?

_____ Days per week

How much time did you usually spend **sitting** while consuming meals on one of those days?

___ ___ Hours per day

___ ___ ___ Minutes per day

3. GENERAL INFORMATION

What is your age? ___

What is your gender? ___

How would you describe your family situation?

O Married
O Widow/widower
O Being divorced
O Single
O Living with a partner

How many children do you have? ___

How many grandchildren do you have? ___

What is your highest achieved diploma or certificate?

O Primary school
O Vocational secondary education
O Technical secondary education
O General secondary education
O Higher education, non-university
O Higher education, university
