# Supplementary material for: Sex-specific typologies of older adults’ sedentary behaviors and their associations with health-related and socio-demographic factors: a latent profile analysis
Source: BMC Geriatr. 2021 Jan 19;21:66. doi: 10.1186/s12877-021-02011-5 (PMC7816402; doi:10.1186/s12877-021-02011-5)
Supplement: Supplementary file 2 — Additional file 2. English language translation of the Busschaert questionnaire [file 12877_2021_2011_MOESM2_ESM.pdf]

Busschaert Questionnaire for 65-years or older

A) General information

Some general questions

Anonymous processing!!

- Initials first name and last name: ______________________
- Date of birth (day/month/year): ☐☐/☐☐/☐☐☐☐
- Today's date (day/month/year): ☐☐/☐☐/☐☐☐☐
- E-mail address (if possible) ______________________
- Phone number or mobile phone number (only used if information is unclear) ☐☐☐☐/☐☐☐☐☐☐

A1) Gender?

☐ Man ☐ Woman

A2) How would you describe your family situation?

☐ Single

☐ Having a partner, but living independently

☐ Living with a partner

☐ Married

☐ Widow/widower

A3) Do you have (grand) children?

☐ Yes

☐ No

A4) In what environment do you live?

☐ Countryside

☐ Village or town

☐ City suburb

☐ City

A5) Have you moved to another address the last year?

☐ Yes ☐ No

A6) Are you hindered by standing upright? (e.g. a recent operation, back pain, …)?

☐ Yes, I cannot stand upright

☐ No, I can stand upright

Sitting and light intense activities

B) Watching television (TV) in leisure time

B1) During the last 7 days, how much time did you spend on average a day sitting/lying while watching TV in leisure time? Answer these questions separately for a weekday and weekend day.

**WATCHING TELEVISION**	
Weekday	Weekend day
☐ Not	☐ Not
☐ 1 to 15 minutes/day	☐ To 15 minutes/day
☐ 15 to 30 minutes/day	☐ 15 to 30 minutes/day
☐ 30 to 60 minutes/day	☐ 30 to 60 minutes/day
☐ 1 to 2 hours/day	☐ 1 to 2 hours/day
☐ 2 to 3 hours/day	☐ 2 to 3 hours/day
☐ 3 to 4 hours/day	☐ 3 to 4 hours/day
☐ 4 to 5 hours/day	☐ 4 to 5 hours/day
☐ 5 to 6 hours/day	☐ 5 to 6 hours/day
☐ 6 to 7 hours/day	☐ 6 to 7 hours/day
☐ More than 7 hours/day	☐ More than 7 hours/day

Please indicate with the use of the reply-card (1) the extent to which you agree with each of the following statements.

	Strongly disagree	Somewhat disagree	Neutral	Somewhat agree	Strongly agree	Not applicable
B2) I think watching TV is pleasant	☐	☐	☐	☐	☐	☐
B3) Watching TV takes time away from doing other important things	☐	☐	☐	☐	☐	☐
B4) I enjoy watching TV for many hours a time	☐	☐	☐	☐	☐	☐
B5) Watching TV is my way to relax	☐	☐	☐	☐	☐	☐
B6) My partner thinks I spend too much time watching TV	☐	☐	☐	☐	☐	☐
B7) I think that I spend too much time watching TV	☐	☐	☐	☐	☐	☐
B8) I consider it possible to reduce my TV time	☐	☐	☐	☐	☐	☐
B9) I consider it possible to turn off the TV during weekend days until 5:00 p.m.	☐	☐	☐	☐	☐	☐
B10) I consider it possible to turn off the TV during meals	☐	☐	☐	☐	☐	☐
B11) My partner encourages me to watch less TV	☐	☐	☐	☐	☐	☐
B12) My friends encourage me to watch less TV	☐	☐	☐	☐	☐	☐

B13) I think elderly people better do <u>not</u> watch more than...

☐ 30 minutes TV per day

☐ 1 hour TV per day

☐ 2 hours TV per day

☐ 3 hours TV per day

☐ 4 hours TV per day

☐ 5 hours TV per day

B14) In which room is the TV that you watch the most?

☐ In the kitchen

☐ In the living room

☐ In a separate room

☐ In the bedroom

☐ In another room (note): ______________________________

B15) How long do your partner watch TV in <u>leisure time</u>?

☐ Not applicable	☐ 2 to 3 hours/day
☐ Does not watch TV	☐ 3 to 4 hours/day
☐ 1 to 15 minutes/day	☐ 4 to 5 hours/day
☐ 15 to 30 minutes/day	☐ 5 to 6 hours/day
☐ 30 to 60 minutes/day	☐ 6 to 7 hours/day
☐ 1 to 2 hours/day	☐ More than 7 hours/day

B16) How often do you watch TV TOGETHER with your family members in <u>leisure time</u>?

☐ Not applicable

☐ Never

☐ Seldom

☐ Sometimes

☐ Often

☐ Very often

There are several situations where you can reduce or (more) interrupt sedentary behaviour while watching TV by standing up for a moment. Indicate for each statement to which extent you would consider it possible to execute the following tasks.

	Strongly disagree	Somewhat disagree	Neutral	Somewhat agree	Strongly agree
B17) To implement standing tasks while watching TV? (e.g. ironing, doing chores, easy playing with the (grand)children,…)	☐	☐	☐	☐	☐
B18) To replace watching TV for 1 hour by standing tasks with light effort (e.g. cleaning, doing chores)?	☐	☐	☐	☐	☐
B19) To stand up (short bouts) during advertisements while watching TV?	☐	☐	☐	☐	☐

C) THE USE OF COMPUTER IN LEISURE TIME

C1) During the last 7 days, how much time did you spend on average a day sitting/lying while using the computer in leisure time? Answer this question separately for a weekday and weekend day.

COMPUTERUSE IN LEISURE TIME	
Weekday	Weekend day
☐ Not	☐ Not
☐ 1 to 15 minutes/day	☐ To 15 minutes/day
☐ 15 to 30 minutes/day	☐ 15 to 30 minutes/day
☐ 30 to 60 minutes/day	☐ 30 to 60 minutes/day
☐ 1 to 2 hours/day	☐ 1 to 2 hours/day
☐ 2 to 3 hours/day	☐ 2 to 3 hours/day
☐ 3 to 4 hours/day	☐ 3 to 4 hours/day
☐ 4 to 5 hours/day	☐ 4 to 5 hours/day
☐ 5 to 6 hours/day	☐ 5 to 6 hours/day
☐ 6 to 7 hours/day	☐ 6 to 7 hours/day
☐ More than 7 hours/day	☐ More than 7 hours/day

C2) How long on average does your partner use the computer (think about tablets, internet on smartphone, laptop, computer,…) in <u>leisure time</u>?

☐ Not applicable	☐ 2 to 3 hours/day
☐ Doesn't use computer	☐ 3 to 4 hours/day
☐ 1 to 15 minutes/day	☐ 4 to 5 hours/day
☐ 15 to 30 minutes/day	☐ 5 to 6 hours/day
☐ 30 to 60 minutes/day	☐ 6 to 7 hours/day
☐1 to 2 hours/day	☐ More than 7 hours/day

C3) How often do you use the computer (seated) at the same moment with your family members using a different computer in the same room in leisure time?

- ☐ Never
- ☐ Seldom
- ☐ Sometimes
- ☐ Often
- ☐ Very often

There are several situations where you can reduce or (more) interrupt sedentary behaviour during the use of a computer by standing up for a moment. Indicate for each statement with the use of the reply-card (2) to which extent you would consider it possible to execute the following tasks.

	Strongly disagree	Somewhat disagree	Neutral	Somewhat agree	Strongly agree
C4) to stand up while using a computer?	☐	☐	☐	☐	☐
C5) to replace using a computer for 1 hour by standing tasks with light effort (e.g. cleaning, doing chores)?	☐	☐	☐	☐	☐
C6) to stand up for a couple of minutes after using a computer while sitting for 30 minutes?	☐	☐	☐	☐	☐

Indicate with the use of the reply-card (1) for each statement about the use of the computer in leisure time to which extent you agree with it

	Strongly disagree	Somewhat disagree	Neutral	Somewhat agree	Strongly agree	Not applicable
C7) I think using a computer is pleasant	☐	☐	☐	☐	☐	☐
C8) Using a computer takes time away from doing other important things	☐	☐	☐	☐	☐	☐
C9) I enjoy using a computer for many hours at a time	☐	☐	☐	☐	☐	☐
C10) Using a computer is my way to relax	☐	☐	☐	☐	☐	☐
C11) My partner thinks I spend too much time using a computer	☐	☐	☐	☐	☐	☐
C12) I think that I spend too much time using a computer in leisure time	☐	☐	☐	☐	☐	☐
C13) I consider it possible that I do not use a computer for some days in the week (leisure time)	☐	☐	☐	☐	☐	☐
C14) I consider it possible to reduce my computer time in leisure time	☐	☐	☐	☐	☐	☐
C15) My partner encourages me to spend less time using a computer in leisure time	☐	☐	☐	☐	☐	☐
C16) My friends encourage me to spend less time using a computer in leisure time	☐	☐	☐	☐	☐	☐

D) MOTORIZED TRANSPORT AS PART OF COMMUTER TRAFFIC AND IN LEISURE TIME

- With motorized transport we mean: car, moped, motorbike, train, tram, bus.
- With each question there is one of the following circles (show accompanied picture with the circles):

Picture motorized transport

Picture active transport

At the start of this part of the questionnaire, make clear that the picture demonstrates the type of transport the questionnaire is talking about.

- If you never use any of the transport above (as a passenger or driver), then you can continue to page 15: "E) Household tasks and making phone calls".

D1) During the last 7 days, how much time did you spend on average a day sitting while using motorized transport. Here, take into account transport in leisure time (e.g. to the shop, visiting friends,…). Take into account car, bus, train, tram, motorbike or moped; do not take into account cycling.

Picture motorized transport

Weekday	Weekend day
☐ Not	☐ Not
☐ 1 to 15 minutes/day	☐ 1 to 15 minutes/day
☐ 15 to 30 minutes/day	☐ 15 to 30 minutes/day
☐ 30 to 45 minutes/day	☐ 30 to 45 minutes/day
☐ 45 to 60 minutes/day	☐ 45 to 60 minutes/day
☐ 60 to 90 minutes/day	☐ 60 to 90 minutes/day
☐ 90 to 120 minutes/day	☐ 90 to 120 minutes/day
☐ 2 to 2.5 hours/day	☐ 2 to 2.5 hours/day
☐ 2.5 to 3 hours/day	☐ 2.5 to 3 hours/day
☐ 3 to 4 hours/day	☐ 3 to 4 hours/day
☐ 4 to 5 hours/day	☐ 4 to 5 hours/day
☐ 5 to 6 hours/day	☐ 5 to 6 hours/day
☐ 6 to 7 hours/day	☐ 6 to 7 hours/day
☐ More than 7 hours/day	☐ More than 7 hours/day

D2) How much time of your answer above were you the driver of a motorized vehicle (moped, motorbike or car)?

Picture motorized transport

Weekday	Weekend day
☐ Not	☐ Not
☐ 1 to 15 minutes/day	☐ 1 to 15 minutes/day
☐ 15 to 30 minutes/day	☐ 15 to 30 minutes/day
☐ 30 to 45 minutes/day	☐ 30 to 45 minutes/day
☐ 45 to 60 minutes/day	☐ 45 to 60 minutes/day
☐ 60 to 90 minutes/day	☐ 60 to 90 minutes/day
☐ 90 to 120 minutes/day	☐ 90 to 120 minutes/day
☐ 2 to 2.5 hours/day	☐ 2 to 2.5 hours/day
☐ 2.5 to 3 hours/day	☐ 2.5 to 3 hours/day
☐ 3 to 4 hours/day	☐ 3 to 4 hours/day
☐ 4 to 5 hours/day	☐ 4 to 5 hours/day
☐ 5 to 6 hours/day	☐ 5 to 6 hours/day
☐ 6 to 7 hours/day	☐ 6 to 7 hours/day
☐ More than 7 hours/day	☐ More than 7 hours/day

D3) Please indicate how much time you spent on average a day walking outdoors at a calm pace (e.g.: wandering = easy walking, not hurried) as part of your transport (for example walking to work, visiting someone, walking to the bus, walking to the shop,…) during the last 7 days.

Picture active transport

Weekday	Weekend day
☐ Not	☐ Not
☐ 1 to 15 minutes/day	☐ 1 to 15 minutes/day
☐ 15 to 30 minutes/day	☐ 15 to 30 minutes/day
☐ 30 to 45 minutes/day	☐ 30 to 45 minutes/day
☐ 45 to 60 minutes/day	☐ 45 to 60 minutes/day
☐ 1 to 2 hours/day	☐ 1 to 2 hours/day
☐ More than 2 hours/day	☐ More than 2 hours/day

Picture motorized transport

Indicate with the use of your reply-card (1) for each statement about the use of motorized transport to which extent you agree with them

	Strongly disagree	Somewhat disagree	Neutral	Somewhat agree	Strongly agree	Not applicable
D4) I think using motorized transport is pleasant	☐	☐	☐	☐	☐	☐
D5) I think it is pleasant to work or to rest as a passenger during motorized transport	☐	☐	☐	☐	☐	☐
D6) I feel lazy arriving at my destination after motorized transport	☐	☐	☐	☐	☐	☐
D7) I think that I spend too much time using motorized transport	☐	☐	☐	☐	☐	☐
D8) I consider it possible to get off the bus/metro spontaneously 1 stop before my destination and to walk the remaining distance	☐	☐	☐	☐	☐	☐
D9) I consider it possible to park the car somewhat further spontaneously and to walk the remaining distance	☐	☐	☐	☐	☐	☐
D10) I consider it possible to take the bicycle or to go by foot spontaneously even if it is possible to use a car	☐	☐	☐	☐	☐	☐
D11) My partner encourages me to use (more often) active transport (to bicycle or to walk)	☐	☐	☐	☐	☐	☐
D12) My friends encourage me to use (more often) active transport (to bicycle or to walk)	☐	☐	☐	☐	☐	☐
D13) My partner thinks I spend too much time using motorized transport	☐	☐	☐	☐	☐	☐

Picture active transport

D14) What are your primary reasons not to participate in active transport (walking or cycling)?

☐ The environment is not safe for walking or bicycling

☐ Active transport (walking or cycling) is not attractive to me (no green environment, boring,…)

☐ Active transport is exhausting (sweating, carrying luggage,…)

☐ Well-developed services for public transport (nearby bus stops,…)

☐ Time pressure

☐ Too great distance

☐ Weather conditions

☐ Combining several tasks (going to the bakery, dropping the children,…)

☐ Financial reasons (repayment public transport, mark-down over 65s)

☐ Other (note): ____

____

☐ I always use active transport (walking and/or cycling)

D15) The most chosen transportation possibility of your partner…

☐ Motorized transport

☐ Active transport (walking/cycling)

☐ Not applicable

Picture motorized transport

There are several situations where you can reduce or (more) interrupt sedentary behaviour during motorized transport by standing up for a moment. Indicate for each statement with the use of your reply-card (2) to which extent you would consider it possible to execute the following tasks.

	Strongly disagree	Somewhat disagree	Neutral	Somewhat agree	Strongly agree
D16) to stand up spontaneously in a bus, train or metro (instead of sitting)?	☐	☐	☐	☐	☐
D17) to stand up (for a short duration) after sitting for 30 minutes in bus, train or metro?	☐	☐	☐	☐	☐

Give your opinion on the following statements related to the environment where you travel during motorized transport. Assess the area within approximately one kilometer of your home or that you could walk to in 10-15 minutes.

Picture motorized transport

	Strongly disagree	Somewhat disagree	Neutral	Somewhat agree	Strongly agree
D18) It is easy to park the car at public places (shops, station,…)	☐	☐	☐	☐	☐
D19) I receive a lot of information about 'sitting activities' like cinema or sport games via billboards along the road and/or the radio	☐	☐	☐	☐	☐

D20) How often do you use motorized transport together with your partner?

Picture motorized transport

**Weekday**	**Weekend day**
☐ Not applicable	☐ Not applicable
☐ Never	☐ Never
☐ Seldom	☐ Seldom
☐ Sometimes	☐ Sometimes
☐ Often	☐ Often
☐ Very often	☐ Very often

E) Household tasks and making phone calls

E1) What is the average time per day you spent the last 7 days on household tasks and making phone calls while sitting? Possible household tasks are: cooking, ironing, making small repairs,…)

**Household tasks (cooking, repairing,…) performed while being seated**		**Phone calls performed while being seated**	
**Weekday**	**Weekday**	**Weekend day**	**Weekend day**
☐ Not	☐ Not	☐ Not	☐ Not
☐ 1 to 15 minutes/day	☐ 1 to 15 minutes/day	☐ To 15 minutes/day	☐ To 15 minutes/day
☐ 15 to 30 minutes/day	☐ 15 to 30 minutes/day	☐ 15 to 30 minutes/day	☐ 15 to 30 minutes/day
☐ 30 to 60 minutes/day	☐ 30 to 60 minutes/day	☐ 30 to 60 minutes/day	☐ 30 to 60 minutes/day
☐ 1 to 2 hours/day	☐ 1 to 2 hours/day	☐ 1 to 2 hours/day	☐ 1 to 2 hours/day
☐ 2 to 3 hours/day	☐ 2 to 3 hours/day	☐ 2 to 3 hours/day	☐ 2 to 3 hours/day
☐ 3 to 4 hours/day	☐ 3 to 4 hours/day	☐ 3 to 4 hours/day	☐ 3 to 4 hours/day
☐ 4 to 5 hours/day	☐ 4 to 5 hours/day	☐ 4 to 5 hours/day	☐ 4 to 5 hours/day
☐ 5 to 6 hours/day	☐ 5 to 6 hours/day	☐ 5 to 6 hours/day	☐ 5 to 6 hours/day
☐ 6 to 7 hours/day	☐ 6 to 7 hours/day	☐ 6 to 7 hours/day	☐ 6 to 7 hours/day
☐ More than 7 hours/day	☐ More than 7 hours/day	☐ More than 7 hours/day	☐ More than 7 hours/day

The following statements are different opinions about sitting during household tasks and making phone calls at home. Please indicate with the use of your reply-card (1) to which extent you agree with the following statements.

	Strongly disagree	Somewhat disagree	Neutral	Somewhat agree	Strongly agree	Not applicable
E2) I think it is pleasant to sit while doing household tasks	☐	☐	☐	☐	☐	☐
E3) I work faster when I sit during household tasks	☐	☐	☐	☐	☐	☐
E4) I would like to stand more often while doing household tasks	☐	☐	☐	☐	☐	☐
E5) I think that I can stand up more often while doing household tasks	☐	☐	☐	☐	☐	☐
E6) I consider it possible to stand up while calling	☐	☐	☐	☐	☐	☐
E7) I consider it possible to stand up while doing tasks in the kitchen	☐	☐	☐	☐	☐	☐
E8) My partner encourages me to sit less while doing household tasks	☐	☐	☐	☐	☐	☐
E9) Households tasks (and making phone calls) are usually done while sitting by my partner	☐	☐	☐	☐	☐	☐

How would you like to perform the following household tasks?

	Standing	Sitting
E10) Making phone calls	☐	☐
E11) Peeling potatoes/cutting vegetables	☐	☐
E12) Ironing	☐	☐
E13) Folding laundry	☐	☐
E14) Brushing teeth	☐	☐
E15) to repair something small	☐	☐

There are several situations where you can reduce or (more often) interrupt sedentary behaviour during household tasks by standing up for a moment. Indicate for each statement with the use of your reply-card (2) to which extent you would consider it possible to execute the following tasks.

	Strongly disagree	Somewhat disagree	Neutral	Somewhat agree	Strongly agree
E16) to do more household tasks while being upright?	☐	☐	☐	☐	☐
E17) to place appliances at a distance so that you would have to stand up to operate them (e.g. printer, remote controller TV,…)?	☐	☐	☐	☐	☐

Please indicate to which extent you agree with the following statements with the use of your reply-card (1).

	Strongly disagree	Somewhat disagree	Neutral	Somewhat agree	Strongly agree	Not applicable
**I am sure that I can interrupt my sitting activities (more often) at home, even if:**						
E18) my partner is not doing this	☐	☐	☐	☐	☐	☐
E19) my partner does not support/understand me	☐	☐	☐	☐	☐	☐

We would like to find out more information about the way that you think about your neighbourhood, home environment and workplace or study environment. Please put one check mark ("X") per answer that best applies to your view of your neighbourhood, home environment and workplace or study environment. By your neighbourhood we mean ALL the area within approximately one kilometer or half a mile of your home or that you could walk to in 10-15 minutes.

	**Yes**	**No**
E20) Most of the houses in my neighbourhood are detached houses	☐	☐
E21) There are many shops within easy walking distance of my home	☐	☐
E22) There is a bus/tram station within easy walking distance of my home	☐	☐
E23) There is a park within easy walking distance of my home	☐	☐
E24) Walking is dangerous because of the traffic in my neighbourhood	☐	☐
E25) Walking is dangerous because of the level of crime in my neighbourhood	☐	☐
E26) There are trees along the streets in my neighbourhood	☐	☐
E27) My neighbourhood is a good place to live in	☐	☐
E28) The environment does not invite to exercise (a lot of hills, no sidewalk,…)	☐	☐
E29) At my home, I have small sports equipment such as a ball, racquets,… for my personal use	☐	☐

How many of the following non-portable electronic devices do you use and are present at your home?

	None	1	2	3	4	5	More than 5
E30) TV's	☐	☐	☐	☐	☐	☐	☐
E31) DVD players/video players	☐	☐	☐	☐	☐	☐	☐
E32) music players (radio, CD player, stereo,…)	☐	☐	☐	☐	☐	☐	☐
E33) computers (desktop)	☐	☐	☐	☐	☐	☐	☐
E34) telephones (dedicated line)	☐	☐	☐	☐	☐	☐	☐

How many of these non-portable electronic devices are present in your bedroom?

	None	1	2	3	4	5	More than 5
E35) TV's	☐	☐	☐	☐	☐	☐	☐
E36) DVD players/video players	☐	☐	☐	☐	☐	☐	☐
E37) music players (radio, CD player, stereo,…)	☐	☐	☐	☐	☐	☐	☐
E38) computers (desktop)	☐	☐	☐	☐	☐	☐	☐
E39) telephones (dedicated line)	☐	☐	☐	☐	☐	☐	☐

How many of the following portable electronic devices do you use and are present at your home?

	None	1	2	3	4	5	More than 5
E40) Laptop	☐	☐	☐	☐	☐	☐	☐
F41) Mobile phones (no smartphone)	☐	☐	☐	☐	☐	☐	☐
F42) Smartphones	☐	☐	☐	☐	☐	☐	☐
F43) Music players (iPod, MP3,…)	☐	☐	☐	☐	☐	☐	☐
F44) Tablet (iPad, Samsung Galaxy Tab,…)	☐	☐	☐	☐	☐	☐	☐

How do you judge the following statements about facilities at your home?

	Strongly disagree	Somewhat disagree	Neutral	Somewhat agree	Strongly agree	Not applicable
E45) The remote controller (TV) can always be found closely to me when I need it	☐	☐	☐	☐	☐	☐
E46) The couches at our place are comfortable to sit for a long time	☐	☐	☐	☐	☐	☐

F) Other sedentary activities

		Not	1 to 15 minutes per day	15 to 30 minutes per day	30 to 60 minutes per day	1 to 2 hours per day	2 to 3 hours per day	3 to 4 hours per day	4 to 5 hours per day	5 to 6 hours per day	6 to 7 hours per day	More than 7 hours per day
**F1) Sitting while reading- (book, magazine, newspaper,…)**	Weekday	☐	☐	☐	☐	☐	☐	☐	☐	☐	☐	☐
	Weekend day	☐	☐	☐	☐	☐	☐	☐	☐	☐	☐	☐
**F2) Sitting while caring for (grand) children, others**	Weekday	☐	☐	☐	☐	☐	☐	☐	☐	☐	☐	☐
	Weekend day	☐	☐	☐	☐	☐	☐	☐	☐	☐	☐	☐
**F3) Sitting for hobbies (voluntary work, playing cards, sewing, Sudoku, crossword,…)**	Weekday	☐	☐	☐	☐	☐	☐	☐	☐	☐	☐	☐
	Weekend day	☐	☐	☐	☐	☐	☐	☐	☐	☐	☐	☐
**F4) Sitting for socializing (visiting friends, in a pub,…)**	Weekday	☐	☐	☐	☐	☐	☐	☐	☐	☐	☐	☐
	Weekend day	☐	☐	☐	☐	☐	☐	☐	☐	☐	☐	☐
**F5) Sitting while listening to music (radio, CD,…)**	Weekday	☐	☐	☐	☐	☐	☐	☐	☐	☐	☐	☐
	Weekend day	☐	☐	☐	☐	☐	☐	☐	☐	☐	☐	☐
**F6) Sitting during meals (breakfast,…)- do NOT include meals while sitting and watching TV**	Weekday	☐	☐	☐	☐	☐	☐	☐	☐	☐	☐	☐
	Weekend day	☐	☐	☐	☐	☐	☐	☐	☐	☐	☐	☐
**F7) Afternoon nap**	Weekday	☐	☐	☐	☐	☐	☐	☐	☐	☐	☐	☐
	Weekend day	☐	☐	☐	☐	☐	☐	☐	☐	☐	☐	☐

How often do you do the following situations at the SAME time (simultaneously)?

	Never	Seldom	Sometimes	Often	Very often	Not applicable
F8) Watching TV AND using mobile phone	☐	☐	☐	☐	☐	☐
F9) Watching TV AND using computer/tablet	☐	☐	☐	☐	☐	☐
F10) Using computer AND using mobile phone	☐	☐	☐	☐	☐	☐
F11) Using computer AND listening to music	☐	☐	☐	☐	☐	☐
F12) Using mobile phone (texting) AND having conversation with friends/family	☐	☐	☐	☐	☐	☐
F13) Using mobile phone AND listening to music	☐	☐	☐	☐	☐	☐
F14) Sewing (or similar tasks) AND watching TV	☐	☐	☐	☐	☐	☐

R) Upright activities

In the last 7 days on average, how much time did you spend during the following upright activities per day? (give reply-card 3)

**Examples of light intense household activities (executed upright)**			
Dusting (A)<br><br>Cooking<br><br>Ironing (A)	Doing the dishes, cleaning the table<br><br>Watering plants<br><br>Making standing phone calls	get (un)dressed<br><br>Playing with the (grand)children (easy)<br><br>Serving dinner	Feeding animals<br><br>Personal care (shaving, make-up,…)<br><br>Doing laundry, hang up clothes to dry,… (A)

		**Not**	**1 to 15 minutes/day**	**15 to 30 minutes/day**	**30 to 45 minutes/day**	**45 to 60 minutes/day**	**1 to 2 hours/day**	**More than 2 hours/day**
**HOUSEHOLD TASKS (executed upright)**								
**R1) Cleaning** (dusting, ironing,…) (A)	weekday	☐	☐	☐	☐	☐	☐	☐
	weekend day	☐	☐	☐	☐	☐	☐	☐
**R2) Kitchen** (cooking, preparations,…)	weekday	☐	☐	☐	☐	☐	☐	☐
	weekend day	☐	☐	☐	☐	☐	☐	☐
**R3) (Grand)children** (playing quietly together,…)	weekday	☐	☐	☐	☐	☐	☐	☐
	weekend day	☐	☐	☐	☐	☐	☐	☐
**Garden (executed upright)**								
R4) Watering plants, feeding animals	weekday	☐	☐	☐	☐	☐	☐	☐
	weekend day	☐	☐	☐	☐	☐	☐	☐

**PERSONAL CARE (executed upright)**								
**R5) Personal care** (washing, shaving, make-up, brushing teeth,…)	weekday	☐	☐	☐	☐	☐	☐	☐
	weekend day	☐	☐	☐	☐	☐	☐	☐
**LEISURE TIME/HOBBIES (executed upright)**								
R6) Shopping, watching TV upright, listening to music upright, light stretching, easy yoga, play pills, standing in the church,…	weekday	☐	☐	☐	☐	☐	☐	☐
	weekend day	☐	☐	☐	☐	☐	☐	☐
**EASY WALKING (e.g. wandering)**								
R7) Walking at a calm pace (e.g. wandering) at home	weekday	☐	☐	☐	☐	☐	☐	☐
	weekend day	☐	☐	☐	☐	☐	☐	☐

G) Health

G1) During the past month, have you often been feeling down, depressed or hopeless?

☐ Yes ☐ No

G2) During the past month, have you had little interest or pleasure in doing things?

☐ Yes ☐ No

H) Physical activity

H1) During the last 7 days, on how many days did you walk for at least 10 minutes at a time? Think about walking at work and home, to get from place to place and in your spare time for recreation, exercise or sport.

☐ No walking → if no walking: go to H2

_day(s) per week

How much time did you usually spend on one of those days where you walked for at least 10 minutes?

_hour(s) minutes per day

H2) How many days did you perform moderate physical activities, like gardening, cleaning (washing windows), easy cycling, easy swimming or other fitness activities during the last 7 days. Please do not include walking. Moderate physical activities refer to activities that take moderate physical effort and make you breathe somewhat harder than normal. Again, think about only those physical activities that you did for at least 10 minutes at a time.

☐ I did not perform moderate physical activity → go to H3

_day(s) per week

How much time did you usually spend on those days where you were moderate physical active?

hour(s) minutes per day

H3) During the last 7 days, on how many days did you do vigorous physical activities like heavy lifting, digging, chopping wood, aerobics, jogging/running or fast cycling? Think about only those physical activities that you did for at least 10 minutes at a time. Vigorous physical activities refer to activities that take hard physical effort and make you breathe much harder than normal.

☐ I did not perform vigorous physical activity → go to I)

_day(s) per week

How much time did you usually spend on those days where you were vigorous physical active?

_hour(s) minutes per day

I) Information section

I1) What is your native language? (Please indicate one answer)

☐ Dutch

☐ French

☐ Turkish

☐ Arabic

☐ English

☐ Other (note): ____
____

I2) What is your highest achieved diploma or certificate?

YOURSELF	PARTNER
☐ Primary school	☐ I don't know
☐ Vocational Secondary Education	☐ Primary school
☐ Technical Secondary Education	☐ Vocational Secondary Education
☐ General Secondary Education	☐ Technical Secondary Education
☐ Higher education, non-university	☐ General Secondary Education
☐ Higher education, university	☐ Higher education, non-university
	☐ Higher education, university

I3) At the moment I have/work/am/do…

☐ Full-time job

☐ Part-time job

☐ Unemployed/job-applicant

☐ Career interruption

☐ Retired

☐ Disabled (whereby the person cannot work)

I4) What is/was your principal profession?

- ☐ Household
- ☐ Education
- ☐ Employee
- ☐ Executive
- ☐ Self-employed (no free profession)
- ☐ Free profession
- ☐ Workman
- ☐ Other (note): ______________________________

I5) How many operational motorized vehicles are there present in the household, even the ones you do not use yourself?

__________ (write down the answer here)

I6) In general, would you say your health is:

- ☐ Excellent
- ☐ Very good
- ☐ Good
- ☐ Fair
- ☐ Poor

I7) Vigorous activities, such as running, lifting heavy objects, participating in strenuous sports,...

- ☐ Yes, limited a lot
- ☐ Yes, limited a little
- ☐ No, not limited at all

I8) Climbing several flights of stairs

☐ Yes, limited a lot

☐ Yes, limited a little

☐ No, not limited at all

During the past 4 weeks, have you had any of the following problems with your work or other regular daily activities as a result of your physical health?

I9) Accomplished less than you would like

☐ Yes

☐ No

I10) were limited in the kind of work or other activities you wanted to accomplish?

☐ Yes

☐ No

During the past 4 weeks, have you had any of the following problems with your work or other regular daily activities as a result of any emotional problems (such as feeling depressed or anxious)?

I11) Accomplished less than you would like

☐ Yes

☐ No

I12) Didn't do work or other activities as carefully as usual

☐ Yes

☐ No

I13) During the past 4 weeks, how much did pain interfere with your normal work (including both work outside the home and housework)?

- ☐ Not at all
- ☐ A little bit
- ☐ Moderately
- ☐ Quite a bit
- ☐ Extremely

These questions are about how you feel and how things have been with you during the last 4 weeks. For each question, please give the answer that comes closest to the way you have been feeling.

I14) Have you felt calm and peaceful?

- ☐ All of the time
- ☐ Most of the time
- ☐ A good bit of the time
- ☐ Some of the time
- ☐ A little bit of the time
- ☐ None of the time

I15) Did you have a lot of energy?

- ☐ All of the time
- ☐ Most of the time
- ☐ A good bit of the time
- ☐ Some of the time
- ☐ A little bit of the time
- ☐ None of the time

I16) Have you been feeling down, sad?

☐ All of the time

☐ Most of the time

☐ A good bit of the time

☐ Some of the time

☐ A little bit of the time

☐ None of the time

I17) During the past 4 weeks, how much of the time has your physical health or emotional problems interfered with your social activities (like visiting friends or family)?

☐ All of the time

☐ Most of the time

☐ A good bit of the time

☐ Some of the time

☐ A little bit of the time

☐ None of the time

I18) How much time are you afraid to fall?

☐ All of the time

☐ Most of the time

☐ A good bit of the time

☐ Some of the time

☐ A little bit of the time

☐ None of the time

I) Body measurements

**Length**	☐☐☐ cm / ☐☐☐ inch	
**Age**	☐☐☐ year	
**Bodyweight**	☐☐☐ kg / ☐☐☐ pounds	
**Waist circumference**	Measurement 1: ☐☐ cm / ☐☐ inch<br>Measurement 2: ☐☐ cm / ☐☐ inch<br>Measurement 3: ☐☐ cm / ☐☐ inch	
**Hand grip strength**	Measurement 1: ☐☐ kg / ☐☐☐pounds<br>Measurement 2: ☐☐ kg / ☐☐☐pounds	
**Repeated chair stands**	☐☐☐ sec<br>☐ participant was not able to perform this measurement	
**Balance**	Most difficult performed position	Duration (sec)
	☐ side by side<br>☐ semi-tandem<br>☐ full tandem	☐☐☐ sec<br>☐☐☐ sec<br>☐☐☐ sec
**Walking 3m**	Time 1: ☐☐☐ sec<br>Time 2: ☐☐☐ sec<br>☐ participant was not able to perform this measurement	
