# Supplementary material for: Sex-specific typologies of older adults’ sedentary behaviors and their associations with health-related and socio-demographic factors: a latent profile analysis
Source: BMC Geriatr. 2021 Jan 19;21:66. doi: 10.1186/s12877-021-02011-5 (PMC7816402; doi:10.1186/s12877-021-02011-5)
Supplement: Supplementary file 3 — Additional file 3. Descriptive statistics broken down by study [file 12877_2021_2011_MOESM3_ESM.docx]

Additional File 3: Descriptive statistics broken down by study

|  | Total (n = 696) | Busschaert study  (n = 258) | BEPAS study  (n = 438) | Significance of difference |
| --- | --- | --- | --- | --- |
| **Socio-demographic characteristics** |  |  |  |  |
| Age: years, mean (SD) | 74.2 (6.2) | 74.0 (6.2) | 74.3 (6.2) | T=0.69, p=0.49^a^ |
| Family situation  % having a partner  % having children | 67.4%  88.5% | 70.0%  90.3% | 65.8%  87.4% | Ꭓ^2^=1.31, p=0.25^b^  Ꭓ^2^=1.36, p=0.24^b^ |
| Educational level  % with college/university degree | 33.2% | 24.3% | 38.4% | Ꭓ^2^=14.37, p<0.001^b^ |
| **Sedentary behaviors** |  |  |  |  |
| Television time: min/day, median (Q1-Q3) | 180.0 (90.0-240.0) | 210.0 (150.0-270.0) | 180.0 (90.0-240.0) | Z=-4.17, p=0.001^c^ |
| Computer time: min/day, median (Q1-Q3) | 2.6 (0 – 60.0) | 7.5 (0.0 – 70.7) | 0.0 (0.0 – 60.0) | Z=-1.05, p=0.29^c^ |
| Transport-related sitting time: min/day, median (Q1-Q3) | 22.5 (8.6 – 38.6) | 22.5 (7.5 – 37.5) | 24.3 (11.4 – 41.4) | Z=-2.28, p=0.02^c^ |
| Sitting for reading: min/day, median (Q1-Q3) | 57.9 (28.9 – 90.0) | 45.0 (22.5 – 90.0) | 60.0 (30.0 – 90.0) | Z=-2.29, p=0.02^c^ |
| Sitting for hobbies: min/day, median (Q1-Q3) | 5.4 (0 – 45) | 17.1 (17.1 – 45.0) | 0.0 (0 – 34.3) | Z=-3.66, p<0.001^c^ |
| Sitting for socializing: min/day, median (Q1-Q3) | 30.0 (8.6 – 60.0) | 41.8 (16.1 – 90.0) | 25.7 (8.0 – 51.4) | Z=-5.09, p<0.001^c^ |
| Sitting for meals: min/day, median (Q1-Q3) | 90 (60.0 – 90.0) | 90.0 (45.0 – 90.0) | 75.0 (60.0 – 90.0) | Z=-3.16, p=0.002^c^ |
| **Health-related outcomes** |  |  |  |  |
| Body mass index: kg/m^2^, mean (SD) | 24.3 (4.6) | 27.8 (4.0) | 22.5 (3.7) | T=283.29, p<0.001^a^ |
| Waist circumference: cm, mean (SD) | 95.6 (13.0) | 96.8 (13.2) | 94.9 (12.9) | T=3.09, p=0.08^a^ |
| Grip strength: kg, mean (SD) | 28.3 (10.8) | 25.2 (9.2) | 30.3 (11.4) | T=29.88, p<0.001^a^ |
| Physical health-related QOL: mean (SD) | 47.6 (9.1) | 43.5 (6.2) | 50.1 (9.7) | T=84.53, p<0.001^a^ |
| Mental health-related QOL score: mean (SD) | 49.1 (8.5) | 47.7 (5.5) | 49.9 (9.8) | T=10.69, p=0.001^a^ |

SD = standard deviation; Q1 – Q3 = quartile 1 – quartile 3. The physical and mental health-related QOL were calculated with the scoring protocol of the SF12. Scores below 50 represent scores below the average in the population, whereas scores above 50 represent scores above the average in the population.^a^ = Independent Samples T-test, ^b^ = Chi-square test, ^c^ =Mann-Whitney U-test
