# Supplementary material for: Sex-specific typologies of older adults’ sedentary behaviors and their associations with health-related and socio-demographic factors: a latent profile analysis
Source: BMC Geriatr. 2021 Jan 19;21:66. doi: 10.1186/s12877-021-02011-5 (PMC7816402; doi:10.1186/s12877-021-02011-5)
Supplement: Supplementary file 4 — Additional file 4. Older adults’ sedentary behaviors by typology - results of the pairwise comparisons [file 12877_2021_2011_MOESM4_ESM.docx]

Older men’s sedentary behavior by typology

|  | Mean difference | Standard error | P-value |
| --- | --- | --- | --- |
| TV time |  |  |  |
| Typology 1 vs 2 | -30.48 | 14.16 | 0.032 |
| Typology 1 vs 3 | -66.09 | 18.73 | <0.001 |
| Typology 1 vs 4 | -64.75 | 19.97 | 0.001 |
| Typology 1 vs 5 | -1.58 | 21.78 | 0.942 |
| Typology 2 vs 3 | -35.61 | 16.81 | 0.035 |
| Typology 2 vs 4 | -34.27 | 18.19 | 0.060 |
| Typology 2 vs 5 | 28.90 | 20.16 | 0.153 |
| Typology 3 vs 4 | 1.33 | 21.94 | 0.952 |
| Typology 3 vs 5 | 64.50 | 23.60 | 0.007 |
| Typology 4 vs 5 | 63.17 | 24.60 | 0.011 |
| Computer time |  |  |  |
| Typology 1 vs 2 | 18.26 | 7.48 | 0.015 |
| Typology 1 vs 3 | 32.51 | 9.89 | 0.001 |
| Typology 1 vs 4 | -1.24 | 10.55 | 0.907 |
| Typology 1 vs 5 | -215.10 | 11.51 | <0.001 |
| Typology 2 vs 3 | 14.25 | 8.88 | 0.110 |
| Typology 2 vs 4 | -19.50 | 9.61 | 0.043 |
| Typology 2 vs 5 | -233.36 | 10.65 | <0.001 |
| Typology 3 vs 4 | -33.75 | 11.59 | 0.004 |
| Typology 3 vs 5 | -247.61 | 12.47 | <0.001 |
| Typology 4 vs 5 | -213.86 | 12.99 | <0.001 |
| Transport-related sitting time |  |  |  |
| Typology 1 vs 2 | 34.69 | 1.79 | <0.001 |
| Typology 1 vs 3 | 41.81 | 2.37 | <0.001 |
| Typology 1 vs 4 | 31.84 | 2.53 | <0.001 |
| Typology 1 vs 5 | 10.37 | 2.76 | <0.001 |
| Typology 2 vs 3 | 7.12 | 1.79 | 0.001 |
| Typology 2 vs 4 | -2.85 | 2.13 | 0.216 |
| Typology 2 vs 5 | -24.33 | 2.30 | <0.001 |
| Typology 3 vs 4 | -9.97 | 2.78 | <0.001 |
| Typology 3 vs 5 | -31.44 | 2.99 | <0.001 |
| Typology 4 vs 5 | -21.48 | 3.11 | <0.001 |
| Sitting for reading |  |  |  |
| Typology 1 vs 2 | 7.27 | 6.53 | 0.266 |
| Typology 1 vs 3 | -10.38 | 8.64 | 0.231 |
| Typology 1 vs 4 | -12.47 | 9.21 | 0.177 |
| Typology 1 vs 5 | -1.50 | 10.05 | 0.881 |
| Typology 2 vs 3 | -17.65 | 7.76 | 0.024 |
| Typology 2 vs 4 | -19.74 | 8.39 | 0.019 |
| Typology 2 vs 5 | -8.77 | 9.30 | 0.346 |
| Typology 3 vs 4 | -2.09 | 10.12 | 0.836 |
| Typology 3 vs 5 | 8.88 | 10.89 | 0.416 |
| Typology 4 vs 5 | 10.97 | 11.35 | 0.334 |
| Sitting for hobbies |  |  |  |
| Typology 1 vs 2 | 2.30 | 2.92 | 0.431 |
| Typology 1 vs 3 | 5.38 | 3.86 | 0.164 |
| Typology 1 vs 4 | -97.12 | 4.11 | <0.001 |
| Typology 1 vs 5 | 3.66 | 4.49 | 0.415 |
| Typology 2 vs 3 | 3.08 | 3.46 | 0.374 |
| Typology 2 vs 4 | -99.41 | 3.75 | <0.001 |
| Typology 2 vs 5 | 1.36 | 4.15 | 0.743 |
| Typology 3 vs 4 | -102.50 | 4.52 | <0.001 |
| Typology 3 vs 5 | -1.72 | 4.86 | 0.724 |
| Typology 4 vs 5 | 100.78 | 5.07 | <0.001 |
| Sitting for socializing |  |  |  |
| Typology 1 vs 2 | 30.13 | 3.76 | <0.001 |
| Typology 1 vs 3 | -40.77 | 4.97 | <0.001 |
| Typology 1 vs 4 | 6.08 | 5.31 | 0.253 |
| Typology 1 vs 5 | 3.98 | 5.79 | 0.492 |
| Typology 2 vs 3 | -70.91 | 4.47 | <0.001 |
| Typology 2 vs 4 | -24.06 | 4.83 | <0.001 |
| Typology 2 vs 5 | -26.16 | 5.36 | <0.001 |
| Typology 3 vs 4 | 46.85 | 5.83 | <0.001 |
| Typology 3 vs 5 | 44.75 | 6.27 | <0.001 |
| Typology 4 vs 5 | -2.10 | 6.53 | 0.748 |
| Sitting for meals |  |  |  |
| Typology 1 vs 2 | 17.47 | 4.65 | <0.001 |
| Typology 1 vs 3 | 4.72 | 6.15 | 0.444 |
| Typology 1 vs 4 | 11.08 | 6.56 | 0.092 |
| Typology 1 vs 5 | 12.96 | 7.15 | 0.071 |
| Typology 2 vs 3 | -12.76 | 5.52 | 0.021 |
| Typology 2 vs 4 | -6.39 | 5.97 | 0.285 |
| Typology 2 vs 5 | -4.51 | 6.62 | 0.496 |
| Typology 3 vs 4 | 6.36 | 7.20 | 0.378 |
| Typology 3 vs 5 | 8.25 | 7.47 | 0.288 |
| Typology 4 vs 5 | 1.89 | 8.08 | 0.816 |

Older women’s sedentary behavior by typology

|  | Mean difference | Standard error | P-value |
| --- | --- | --- | --- |
| TV time |  |  |  |
| Typology 1 vs 2 | -30.81 | 17.15 | 0.172 |
| Typology 1 vs 3 | 37.07 | 12.14 | 0.007 |
| Typology 2 vs 3 | 67.89 | 18.88 | 0.001 |
| Transport-related sitting time |  |  |  |
| Typology 1 vs 2 | -1.18 | 1.72 | 0.772 |
| Typology 1 vs 3 | -35.38 | 1.22 | <0.001 |
| Typology 2 vs 3 | -34.20 | 1.89 | <0.001 |
| Sitting for reading |  |  |  |
| Typology 1 vs 2 | -2.36 | 7.65 | 0.949 |
| Typology 1 vs 3 | -3.22 | 5.42 | 0.824 |
| Typology 2 vs 3 | -0.86 | 8.43 | 0.994 |
| Sitting for hobbies |  |  |  |
| Typology 1 vs 2 | -108.28 | 5.58 | <0.001 |
| Typology 1 vs 3 | -7.06 | 3.95 | 0.058 |
| Typology 2 vs 3 | 101.22 | 4.79 | <0.001 |
| Sitting for socializing |  |  |  |
| Typology 1 vs 2 | -12.73 | 5.58 | 0.060 |
| Typology 1 vs 3 | -9.08 | 3.95 | 0.057 |
| Typology 2 vs 3 | 3.65 | 6.15 | 0.824 |
| Sitting for meals |  |  |  |
| Typology 1 vs 2 | -2.09 | 5.50 | 0.924 |
| Typology 1 vs 3 | 0.17 | 3.89 | 0.999 |
| Typology 2 vs 3 | 2.26 | 6.05 | 0.926 |
