# Supplementary material for: Sex-specific typologies of older adults’ sedentary behaviors and their associations with health-related and socio-demographic factors: a latent profile analysis
Source: BMC Geriatr. 2021 Jan 19;21:66. doi: 10.1186/s12877-021-02011-5 (PMC7816402; doi:10.1186/s12877-021-02011-5)
Supplement: Supplementary file 5 — Additional file 5. Differences in health-related outcomes and socio-demographics depending on typology – results of the pairwise compairsons [file 12877_2021_2011_MOESM5_ESM.docx]

Differences in health-related outcomes and socio-demographics depending on typology (men)

|  | Mean difference | Standard error | P-value |
| --- | --- | --- | --- |
| BMI |  |  |  |
| Typology 1 vs 2 | -0.03 | 0.65 | 0.97 |
| Typology 1 vs 3 | -1.38 | 0.86 | 0.11 |
| Typology 1 vs 4 | -0.95 | 0.90 | 0.29 |
| Typology 1 vs 5 | 0.03 | 0.97 | 0.97 |
| Typology 2 vs 3 | -1.35 | 0.76 | 0.08 |
| Typology 2 vs 4 | -0.92 | 0.81 | 0.26 |
| Typology 2 vs 5 | 0.06 | 0.90 | 0.95 |
| Typology 3 vs 4 | 0.44 | 0.98 | 0.66 |
| Typology 3 vs 5 | 1.41 | 1.08 | 0.19 |
| Typology 4 vs 5 | -0.98 | 1.10 | 0.37 |
| Waist circumference |  |  |  |
| Typology 1 vs 2 | -0.32 | 1.74 | 0.855 |
| Typology 1 vs 3 | -1.24 | 2.32 | 0.593 |
| Typology 1 vs 4 | 0.43 | 2.41 | 0.858 |
| Typology 1 vs 5 | -1.96 | 2.61 | 0.452 |
| Typology 2 vs 3 | -0.93 | 2.04 | 0.650 |
| Typology 2 vs 4 | 0.75 | 2.14 | 0.730 |
| Typology 2 vs 5 | -1.65 | 2.43 | 0.499 |
| Typology 3 vs 4 | 1.67 | 2.64 | 0.527 |
| Typology 3 vs 5 | -0.72 | 2.89 | 0.803 |
| Typology 4 vs 5 | -2.40 | 2.95 | 0.417 |
| Grip strength |  |  |  |
| Typology 1 vs 2 | 1.80 | 1.44 | 0.215 |
| Typology 1 vs 3 | 2.98 | 1.93 | 0.125 |
| Typology 1 vs 4 | 3.75 | 2.01 | 0.063 |
| Typology 1 vs 5 | -0.90 | 2.17 | 0.679 |
| Typology 2 vs 3 | 1.18 | 1.70 | 0.487 |
| Typology 2 vs 4 | 1.95 | 1.81 | 0.280 |
| Typology 2 vs 5 | -2.69 | 2.02 | 0.184 |
| Typology 3 vs 4 | 0.77 | 2.20 | 0.726 |
| Typology 3 vs 5 | -3.87 | 2.41 | 0.109 |
| Typology 4 vs 5 | 4.65 | 2.45 | 0.059 |
| Physical health-related QOL |  |  |  |
| Typology 1 vs 2 | 2.11 | 1.31 | 0.108 |
| Typology 1 vs 3 | 2.16 | 1.75 | 0.219 |
| Typology 1 vs 4 | 4.76 | 1.82 | 0.009 |
| Typology 1 vs 5 | 2.64 | 1.97 | 0.181 |
| Typology 2 vs 3 | 0.05 | 1.54 | 0.976 |
| Typology 2 vs 4 | 2.65 | 1.64 | 0.107 |
| Typology 2 vs 5 | 0.53 | 1.83 | 0.773 |
| Typology 3 vs 4 | 2.60 | 2.00 | 0.194 |
| Typology 3 vs 5 | 0.48 | 2.18 | 0.825 |
| Typology 4 vs 5 | -2.12 | 2.23 | 0.342 |
| Mental health-related QOL |  |  |  |
| Typology 1 vs 2 | 2.01 | 1.16 | 0.084 |
| Typology 1 vs 3 | 0.71 | 1.55 | 0.647 |
| Typology 1 vs 4 | 0.20 | 1.61 | 0.903 |
| Typology 1 vs 5 | -0.70 | 1.74 | 0.686 |
| Typology 2 vs 3 | -1.30 | 1.36 | 0.341 |
| Typology 2 vs 4 | -1.81 | 1.45 | 0.211 |
| Typology 2 vs 5 | -2.71 | 1.62 | 0.094 |
| Typology 3 vs 4 | 0.52 | 1.76 | 0.771 |
| Typology 3 vs 5 | -1.41 | 1.93 | 0.464 |
| Typology 4 vs 5 | -0.90 | 1.97 | 0.648 |
| Age |  |  |  |
| Typology 1 vs 2 | -1.79 | 0.82 | 0.195 |
| Typology 1 vs 3 | -3.57 | 1.10 | 0.011 |
| Typology 1 vs 4 | -1.28 | 1.16 | 0.806 |
| Typology 1 vs 5 | 2.06 | 1.27 | 0.486 |
| Typology 2 vs 3 | -1.78 | 0.99 | 0.374 |
| Typology 2 vs 4 | 0.51 | 1.06 | 0.989 |
| Typology 2 vs 5 | 3.84 | 1.17 | 0.010 |
| Typology 3 vs 4 | 2.29 | 0.39 | 0.386 |
| Typology 3 vs 5 | 5.62 | 1.39 | 0.001 |
| Typology 4 vs 5 | 3.34 | 1.43 | 0.139 |

Differences in health-related outcomes and socio-demographics depending on typology (women)

|  | Mean difference | Standard error | P-value |
| --- | --- | --- | --- |
| BMI |  |  |  |
| Typology 1 vs 2 | -2.86 | 0.90 | 0.002 |
| Typology 1 vs 3 | -0.12 | 0.62 | 0.843 |
| Typology 2 vs 3 | 2.74 | 0.98 | 0.006 |
| Waist circumference |  |  |  |
| Typology 1 vs 2 | -5.36 | 2.34 | 0.023 |
| Typology 1 vs 3 | -1.41 | 1.63 | 0.387 |
| Typology 2 vs 3 | 3.95 | 2.57 | 0.126 |
| Grip strength |  |  |  |
| Typology 1 vs 2 | 0.66 | 1.05 | 0.534 |
| Typology 1 vs 3 | -1.33 | 0.73 | 0.071 |
| Typology 2 vs 3 | -1.99 | 1.16 | 0.087 |
| Physical health-related QOL |  |  |  |
| Typology 1 vs 2 | 2.05 | 1.69 | 0.228 |
| Typology 1 vs 3 | -2.46 | 1.18 | 0.038 |
| Typology 2 vs 3 | -4.50 | 1.86 | 0.016 |
| Mental health-related QOL |  |  |  |
| Typology 1 vs 2 | 1.85 | 1.72 | 0.281 |
| Typology 1 vs 3 | -1.33 | 1.20 | 0.268 |
| Typology 2 vs 3 | -3.18 | 1.89 | 0.092 |
| Age |  |  |  |
| Typology 1 vs 2 | -0.02 | 1.11 | 1.000 |
| Typology 1 vs 3 | 2.05 | 0.78 | 0.023 |
| Typology 2 vs 3 | 2.07 | 1.22 | 0.206 |
